# Dynamic Reconfiguration and Local Polarization of NiFe‐Layered Double Hydroxide‐Bi_2_MoO_6−_ *_x_* Heterojunction for Enhancing Piezo‐Photocatalytic Nitrogen Oxidation to Nitric Acid

**DOI:** 10.1002/advs.202401667

**Published:** 2024-04-16

**Authors:** Xiaoxu Deng, Peng Chen, Ruirui Cui, Xingyong Gong, Yubo Wu, Xu Wang, Chaoyong Deng

**Affiliations:** ^1^ Key Laboratory of Electronic Composites of Guizhou Province College of Big Data and Information Engineering Guizhou University Guiyang Guizhou 550025 China; ^2^ School of Electronics and Information Engineering Guiyang University Guiyang Guizhou 550005 China; ^3^ Key Laboratory of Green Chemical and Clean Energy Technology of Guizhou Provincial School of Chemistry and Chemical Engineering Guizhou University Guiyang Guizhou 550025 China

**Keywords:** Bi_2_MoO_6_, electronic interaction, interfacial chemical bond, NiFe‐layered double hydroxide, piezo‐photocatalytic nitrogen oxidation to nitric acid

## Abstract

Constructing heterojunctions with vacancies has garnered substantial attention in the field of piezo‐photocatalysis. However, the presence of interfacial vacancies can serve as charge‐trapping sites, leading to the localization of electrons and hindering interfacial charge transfer. Herein, dual oxygen vacancies in the NiFe‐layered double hydroxide and Bi_2_MoO_6−_ *_x_* induced interfacial bonds have been designed for the piezo‐photocatalytic N_2_ oxidation to NO_3_^−^. Fortunately, it achieves sensational nitric acid production rates (7.23 mg g^−1^ h^−1^) in the absence of cocatalysts and sacrificial agents, which is 6.03 times of pure Bi_2_MoO_6_ that under ultrasound and light illumination. Theoretical and experimental results indicate that interfacial bonds act as “charge bridge” and “strain center” to break the carrier local effect and negative effects with piezocatalysis and photocatalysis for promoting exciton dissociation and charge transfer. Moreover, the strong electronic interaction of the interfacial bond induces internal reconstruction under ultrasound for promoting the local polarization and adsorption of N_2_, which accelerates the fracture of the N≡N bonds and reduces the activation energy of the reaction. The research not only establishes a novel approach for optimizing the combined effects of piezo‐catalysis and photocatalysis, but also achieves equilibrium between the synergistic impacts of vacancies and heterojunctions.

## Graphical abstract

NiFe‐layered double hydroxide and Bi_2_MoO_6−_ *_x_* heterojunction with dual oxygen vacancies and interfacial bonds is designed for enhancing piezo‐photocatalytic nitrogen oxidation to nitric acid. The interfacial bonds act as “charge bridge” and “strain center” to promote exciton dissociation and charge transfer as well internal reconstruction for molecular activation.

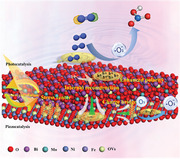

## 1 Introduction

Photocatalytic oxidatively fixing N_2_ with O_2_ and water into nitrate (PNN) holds a promising alternative to the energy‐intensive and multi‐step industrial Ostwald process for HNO_3_ production.^[^ ^1^ ^−^ ^4^ ^]^ However, the high cleavage energy barrier of N≡N triple bonds, the symmetry forbidden molecule orbital of N_2_ and O_2_, and the poor charge transfer rate, result in a sluggish kinetic consideration for the PNN.^[^ ^5^ ^]^ Currently, the strain‐induced piezoelectric field has been found to be a promising and efficient strategy for enhancing photocatalytic performance.^[^ ^6^ ^−^ ^9^ ^]^ It can efficiently modulate the charge migration from the inside to the surface of catalysts, as well as reduce the reaction energy barrier.^[^ ^10^, ^11^ ^]^ Therefore, it is expected that piezo‐photocatalytic oxidatively of N_2_ with O_2_ and water into nitrate (PPNN) can achieve excellent activity.

Various typical semiconductors have been discovered to have ultrahigh efficiency for converting light irradiation and mechanical energy, which include CdS, ZnO, Bi_4_Ti_3_O_12_, Bi_4_NbO_8_, C_3_N_4_ etc.^[^ ^12^ ^−^ ^16^ ^]^ Bismuth molybdate (Bi_2_MoO_6_), a famous member of the Aurivillius‐related oxide family, including intergrowth of [Bi_2_O_2_]^2+^ sheets and perovskite‐like [MoO_4_]^2−^ slabs, is a potential catalyst for piezocatalytic or photocatalytic field.^[^ ^17^ ^−^ ^20^ ^]^ For example, Dong et al. prepared the 2D piezoelectric Bi_2_MoO_6_ for glutathione‐enhanced sonodynamic therapy, which derived from piezoelectric polarization and band tilting to accelerate toxic reactive oxygen species production.^[^ ^21^ ^]^ Ji et al. introduced an ultrathin Bi_2_MoO_6_ catalyst for enhancing the piezo‐photocatalytic elimination of Rhodamine B via producing a built‐in piezoelectric field to drive charge transfer.^[^ ^22^ ^]^ However, like most reported single materials, Bi_2_MoO_6_ possesses the drawback of huge electron transfer resistance and poor activation sites.^[^ ^23^, ^24^ ^]^ Fortunately, appropriately modifying the heterojunctions is the most likely to overcome those difficulties.^[^ ^25^ ^]^ Notably, layered double hydroxides, which have a unique layered structure of cation layer and anion interlayer as well as active sites, are regarded as ideal piezo‐photocatalysts and the optimal alternative for constructing heterojunctions.^[^ ^26^, ^27^ ^]^ However, this strategy requires tight contact at the phase interfaces, which severely hampers further enhancement of the piezo‐photocatalytic performance. On the other hand, vacancies often coexist in isolated materials and act as the charge trapping site and polarization center.^[^ ^28^ ^]^ For heterojunctions, the presence of interfacial vacancies may serve as charge trapping sites, leading to the localization of electrons and hindering interfacial charge transfer. Therefore, a method to balance the piezo‐photocatalytic performance with vacancies and heterojunctions remains a challenging task.

Bear this in mind, the oxygen defect reached NiFe‐layered double hydroxide‐Bi_2_MoO_6−_ *_x_* heterojunctions (BNF‐x) was successfully constructed via a facile hydrothermal method. It is found that the BNF‐4 exhibited an outstanding HNO_3_ yield of 7.23 mg g^−1^ h^−1^ as well as good recycling stability using simulated water, N_2,_ and O_2_ as reactive material under visible light irradiation. Density functional theory (DFT) combined with COMSOL simulation and experimental data revealed that dual oxygen vacancies in the NiFe‐layered double hydroxide and Bi_2_MoO_6−_ *_x_* induced interfacial bonds, which acted as “charge bridge” and “strain center” to boost the charge separation from the heterojunctions and built‐in electric field. On the other hand, the strong electronic interaction in the interfacial bond led to induced internal reconstruction, which accelerated the fracture of the N≡N bonds and reduced the activation energy of the reaction. Our work provides an in‐depth mechanistic insight into the relationship of the ingenious construction of internal structure for regulating charge migration path in catalyst, as well as material design encompasses the fabrication of heterojunctions or the regulation of surface/interface properties.

## 2 Results and Discussion

### 2.1 Morphology and Structure Analysis

**Figure 1:**
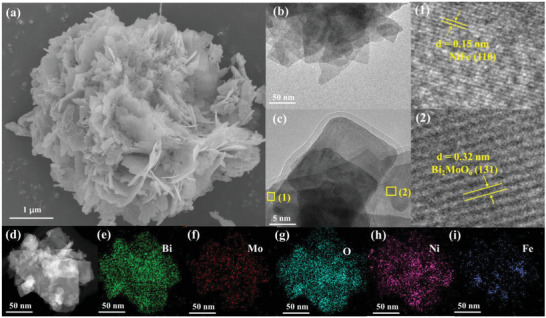
a) SEM image, b–d) TEM, and e–i) the corresponding mapping images of BNF‐4.

The detailed morphology information of the prepared samples was obtained by SEM, TEM, and HRTEM. As presented in **Figure** **1**a and Figure S1 (Supporting Information), all of the synthetic catalyst displayed a nanosheet‐like morphology. The thickness of the prepared samples was explored by the AFM. Notably, the average stacking thickness of BMO, NF, BBNF, and BNF‐4 were estimated to be ≈7.92, 7.94, 20.92, and 16.31 nm (Figure S2, Supporting Information), respectively. Those ultrathin nanosheets contributed to exposing more active sites, enhancing carrier transport, and providing polarization strong in‐plane.^[^ ^29^ ^]^ As shown in Figure 1c, the lattice fringe of 0.32 and 0.15 nm were related to the (131) and (110) crystal planes of the standard Bi_2_MoO_6_ and NiFe LDH,^[^ ^30^, ^31^ ^]^ respectively. The corresponding elemental mappings (Figure 1d–i) revealed that the existence and uniform distribution of Bi, Mo, O, Ni, and Fe elements in BNF‐4, confirming the NiFe‐layered double hydroxide‐Bi_2_MoO_6_ heterojunction was successfully constructed. Moreover, the BET surface areas of BMO, NF, BBNF, and BNF‐4 were 10.6, 19.0, 21.6, and 78.1 m^2^ g^−1^ (Figure S3, Supporting Information), respectively, suggesting that BNF‐4 possesses the highest surface area to expose more active sites for PPNR.

As shown in Figure S4 (Supporting Information), all the prepared composite samples have the characteristic peaks of orthorhombic Bi_2_MoO_6_ and NiFe LDH, implying that the Bi_2_MoO_6_‐NiFe LDH heterojunctions (BNF) was successfully constructed. Notably, compared with BMO and BBNF, the (200) and (002) peaks of BNF shifted toward higher angles, assigning to lattice distortion of BNF and resulted in the reduction of lattice spacing. The lattice distortion in BNF‐4 is further explored by the electron paramagnetic resonance (EPR) spectroscopy. Figure S5 (Supporting Information) shows that BMO and NF possess the large peaks at the g = 2.003, which are assigned to O vacancies,^[^ ^32^, ^33^ ^]^ suggesting that there are O vacancies in the BMO, NF. Compared with the BMO, NF, and BBNF, the highest peak intensity can be seen in BNF‐4, which belongs to the further aggravation of O vacancies, proving dual O vacancies in BMO and NF could further distort the structure of the heterojunction. As displayed in Figure S6 (Supporting Information), the vibration peak of BMO at 144 and 289 cm^−1^ corresponded to specified as the lattice mode of Bi^3+^ and the E_g_ bending of Bi_2_MoO_6_.^[^ ^34^, ^35^ ^]^ Those peaks located at 352 and 401 cm^−1^, 709, 789, and 841 cm^−1^, which belong to the E_u_ symmetric bending and stretching modes of MoO_6_ octahedrons.^[^ ^36^, ^37^ ^]^ Moreover, the peaks at 321, 460, and 534 cm^−1^ are related to the O‐H, Ni‐O, and Fe‐O vibrations in LDH, respectively.^[^ ^38^, ^39^ ^]^ Notably, compared with BMO, NF, and BBNF, the lattice mode of Bi‐O bonds in the BNF‐4 shifted to the lower wavenumber, and the intensity was increased, suggesting the amount and bond length of the Bi‐O bonds are increased. Combined with the EPR and XRD results, it can be concluded that the dual O vacancies induced the interfacial bonds via the formation of heterojunction, and resulted in more structural distortion and interplanar spacing decreased in the BNF‐4. Above all, the O vacancies in Bi_2_MoO_6_ and NiFe LDH induced the interfacial bonds via the formation of heterojunction.

**Figure 2:**
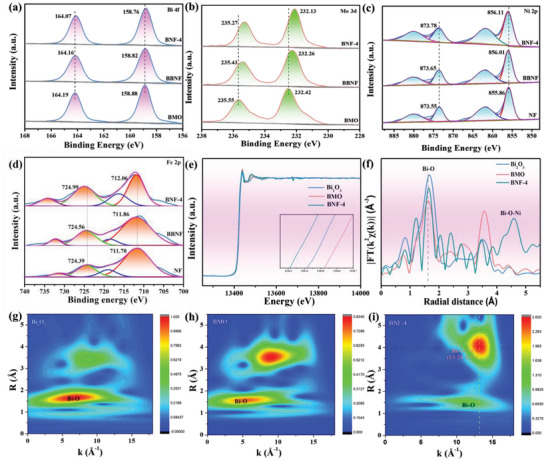
XPS spectra of a) Bi 4f, b) Mo 3d, c) Ni 2p, and d) Fe 2p for prepared samples. e) The normalized XANES spectra of Bi in Bi_2_O_3_, BMO, and BNF‐4. f) Fourier‐transformed Bi K‐edge EXAFS in Bi_2_O_3_, BMO, BNF‐4. Wavelet transformed EXAFS of g) Bi_2_O_3_, h) BMO, and i) BNF‐4.

Due to the formation of interfacial bonds, significant changes may occur for the chemical environment. As shown in **Figure** **2**a, two binding energies of BNF‐4 at 164.07, and 158.76 eV in BNF‐4 are Bi 4f_5/2_ and Bi 4f_7/2_,^[^ ^40^ ^]^ respectively. Two characteristic peaks in BNF‐4 at 232.13 and 235.27 eV are attributed to 3d_5/2_ and 3d_3/2_ of Mo,^[^ ^41^ ^]^ Compared with BMO and BBNF, the negative shift of BNF‐4 in Bi and Mo spectrum can be assigned to the decrease electron cloud around Bi atom, suggesting that the formation of heterojunction strengthened the configuration of O vacancies. As shown in Figure S7 (Supporting Information), the deconvoluted O 1s spectra of BNF‐4 at 529.71, 531.05, and 531.62 eV are coordinated to the presence of metal‐oxygen bonds, defective oxygen and adsorbed oxygen.^[^ ^42^ ^]^ Notably, the ratio of adsorbed oxygen is greatly enhanced, implying the strong enchantment of its O adsorption ability. As presented in Figure 2c, the Ni 2p spectrum can be deconvoluted to four peaks at 856.11, 861.69, 873.78, and 880.11 eV, which are ascribed to the Ni 2p_1/2_, satellite, and Ni 2p_3/2_ peak,^[^ ^43^ ^]^ respectively. Four peaks at 712.06, 716.44, 724.99, and 734.28 eV in BNF‐4 are corresponded to Fe 2p_1/2_, satellite, and Fe 2p_3/2_.^[^ ^44^ ^]^ Compared with NF, the Ni 2p and Fe 2p in BNF‐4 are shifted to higher energies, indicating the electron transfer from the BMO to the NF. Moreover, those results mean that the strong electronic interaction in the interfacial bond. To further understand the contributions of electronic interaction, the density of states (DOS) is calculated. As shown in Figure S8 (Supporting Information), the Bi p orbital overlaps with the O p, suggesting the strong electronic interaction in the BNF via the formation of Bi‐O bonds. Moreover, these phenomena are more obvious and closer to Fermi level under 100 MPa pressure, suggesting that the interfacial bonds further regulate the internal structure under ultrasound.

To make clear the interfacial bonds of local atomic coordination and electronic structure, the X‐ray absorption spectroscopy (XAS) analysis has been carried out. The Bi K‐edge X‐ray absorption near edge structure (XANES) spectra exhibited BNF‐4 shifts to the negative energy compared with BMO, proving a partial reduction of Bi atoms. As shown in Figure 2f, the peaks located at ≈1.65 for BNF‐4 belong to the characteristic of Bi‐O bonds, which is longer than that of Bi‐O bond in BMO, suggesting the interfacial Bi‐O bonds were formed. The coordinate system can be recorded by the full‐range wavelet transforms representation of the EXAFS signal. As exhibited in Figure 2g–i, the Bi‐O‐Ni bond in pure BNF‐4 is located at (13.24, 4.12), confirming the formation of interfacial Bi‐O‐Ni bonds.^[^ ^45^ ^]^ Above all, the interfacial Bi‐O‐Ni bonds were formed in BNF‐4 and resulted in the strong electronic interaction and low coordination atom for the interfacial bond.

### 2.2 Interfacial Bonds Calibrated Piezo‐Photoinduced Charge Transfer

The optical properties of the catalyst were investigated via UV–vis diffuse reflectance spectroscopy (UV–vis DRS). As displayed in Figure S9 (Supporting Information), the absorption edge of BMO and NF are ≈480 and 600 nm, respectively. The absorption edge of BNF heterojunction increases sharply with increasing NF content, which is within the range of 480–600 nm. Accordingly, the energy band values of BMO and NF are computed to be 2.58 and 2.16 eV, respectively (Figure S9b, Supporting Information). As exhibited in Figure S9c (Supporting Information), the VB potential of BMO and NF were 2.41 and 1.81 eV, and their CB potential were determined to be −0.17 and −0.35 eV, respectively. Therefore, it can be deduced that the BNF have an ability to oxidize N_2_ into HNO_3_.^[^ ^46^ ^]^ Based on the above analysis, the photocatalytic generation of **·**OH experiments by the holes (1.99  eV vs NHE) over the prepared samples were further employed to identify the type of heterojunction.^[^ ^47^ ^]^ As shown in Figure S10 (Supporting Information), there are no **·**OH evolution was observed in NF, which is attributed to the VB of the NF is more negative than E(H_2_O/**·**OH). However, BNF‐4 and BBNF both exhibited excellent **·**OH generation performance compared to that of BMO. Therefore, the type II heterojunction mechanism is impossible to be established for BNF‐4, and the direct S‐scheme mechanism can be anticipated in BNF‐4. To further explore the heterojunction type, work functions calculations were performed. As shown in Figure S11 (Supporting Information), the work functions of BMO and NF are 5.90 eV and 6.51 eV, respectively. Consequently, the electrons in Bi_2_MoO_6_ will migrate to NiFe LDH until the Fermi level reaches the equilibrium. Moreover, the work function of BMO and NF are 5.08 and 6.41 eV at 100 Pa pressure, respectively, suggesting the charge transfer more easily under pressure. Above all, the charge transfer between Bi_2_MoO_6_ and NiFe LDH follows the S‐scheme heterojunction, and the charge transfer more easily under pressure.

**Figure 3:**
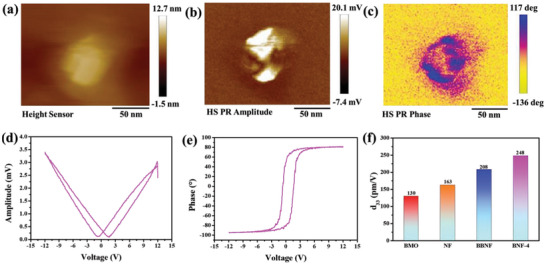
PFM image of BNF‐4: a) topography image, b) amplitude image, c) phase image. d) Piezoresponse amplitude butterfly loops and e) phase hysteresis loops of BNF‐4. f) The maximum effective piezoelectric coefficient of the prepared samples.

In order to obtain the piezoelectric properties, a piezoelectric force microscope (PFM) has been employed. As exhibited in **Figure** **3**a–c and Figure S12 (Supporting Information), the relative amplitude image (Figure S12e–h, Supporting Information) and the phase image (Figure S12i–l, Supporting Information) of all the synthetic catalysts match well with the topographic image, confirming the piezoelectricity characteristic of NiFe‐layered double hydroxide and Bi_2_MoO_6−_ *_x_*. Notably, the clearest relative amplitude image, phase image, and topographic image can be seen in the BNF‐4 (Figure 3a–c), suggesting the significant enhancement of piezoelectricity properties of BNF‐4. Moreover, as shown in Figure 3d,e and Figure S13 (Supporting Information), BNF‐4 exhibits the highest piezoelectric hysteresis curve and butterfly curve, which is harmony with the relative amplitude image results. The maximum effective piezoelectric coefficient of BMO, NF, BBNF, and BNF‐4 are calculated to be 130, 163, 208, and 248 pm V^−1^, respectively, revealing that the dual O vacancies and interfacial bonds enhanced the distortion of structure and result in a larger piezoelectricity. To further investigate the enhanced piezoelectricity of BNF‐4, the COMSOL Multiphysics simulation have been carried out. The models of heterojunction and the pressure arrow the direction were constructed as 50 × 50 × 10 nm size and 10 MPa. As shown in Figures S15 and S16 (Supporting Information), the interfacial bonds models of BNF‐4 have the most obvious charge distribution variance, while the normal heterojunction exhibits uniform charge distribution. On the other hand, the stress center of BNF‐4 mainly locates on the interfacial bonds, while the normal heterojunction has a uniform stress distribution, suggesting that the interfacial bonds could cause larger piezoelectricity. To further confirm those results, we performed the DFT calculation to examine the structural change and charge distribution. As shown in Figure S17 (Supporting Information), the interfacial bonds increase from 2.618 to 2.637 Å under stress, demonstrating that the center of stress may located in the interfacial bonds. Moreover, the charge redistribution occurs in O vacancies and interfacial bonds for BNF‐4, BBNF, NF, and BMO, indicating that the formation of dual O vacancies and interfacial bonds induces strong interfacial polarization. Notably, charge depletion occurs mainly on the O vacancies of NF and BMO for BBNF, which results in a large number of the localized carriers, suggesting the opposite polarization effect for the common heterojunction with vacancies. Strangely, the interfacial bonds induce more negative and positive centers in the heterojunction interfaces, suggesting that the interfacial bonds act as “electron bridge” to promote charge transfer across the S‐scheme. More importantly, those phenomena show more clearly in BNF‐4 under pressure, suggesting that the interfacial bonds exacerbate the uneven charge distribution and act as electron bridge to promote charge transfer under pressure.

**Figure 4:**
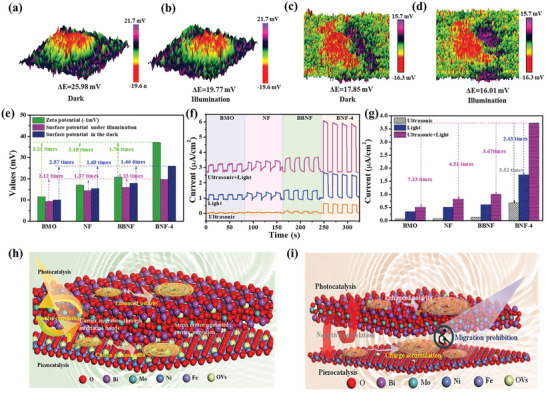
KPFM potential images of BNF‐4: a) in the dark and b) under illumination. BBNF: c) in the dark and d) under illumination. e) The corresponding surface potential and Zeta potential, f,g) Current density of as prepared samples. h,i) Schematic illustration of the photo‐piezocatalytic current process over BNF and BBNF.

Based on the above uneven charge distribution, the internal electric field can be formed. In order to get an in‐depth understanding of the internal electric field, KPFM has been carried out under dark and illumination conditions (Figure S20, Supporting Information). As shown in **Figure** **4**a,b and Figure S20j–l (Supporting Information), BNF‐4 exhibits the highest surface potential of ≈25.98 mV when stressed by the probe tip, suggesting the interfacial bond promotes uneven charge distribution and forms a huge internal electric field. The internal electric field contains two factors: the Zeta potential and the surface potential. As shown in Figure 4e and Figure S21 (Supporting Information), the Zeta potential of BMO, NF, BBNF, and BNF‐4 are −11.56, −17.06, −20.83, and −37.12 mV, respectively. Thus, the internal electric field of BNF‐4 are 3.21, 2.18, and 1.78 times that of BMO, NF, and BBNF, which is conducive to the exciton dissociation and charge transfer. Interestingly, the surface potential of BNF‐4 and BBNF decreases to 19.77 and 16.01 mV under light irradiation, respectively. assigning to statical polarization electric field being screened by the photogenerated carriers, suggesting that a huge inward built‐in electric field is formed and results in the charges moving in a defined direction.^[^ ^48^ ^]^ Compared with BBNF, the interfacial bonds in BNF‐4 greatly weaken the negative effect of electronic localization for promoting charge directional transfer. Therefore, it can be expected that BNF has excellent carrier migration efficiency and exciton separation ability.

As we know, coulomb‐bound electron‐hole pairs play an important role in photocatalysis and piezocatalysis.^[^ ^49^, ^50^ ^]^ Therefore, the steady‐state and transient‐state photoluminescence (PL) measurements are used to illustrate exciton dissociation. As shown in Figure S22 (Supporting Information), BNF‐4 exhibits the slowest fluorescence intensity, suggesting the strongest singlet exciton radiative recombination of BNF‐4. Similarly, the average lifetime of BMO, NF, BBNF, and BNF‐4 are 1.97, 1.60, 1.57, and 0.83 ns, respectively, demonstrating that the dual O vacancies and interfacial bonds provide a huge force to drive the singlet exciton dissociation. Moreover, the exciton dissociation dynamics have been carried out by temperature‐dependent PL spectra (TD‐PL). As shown in Figure S23 (Supporting Information), the estimated E_b_ of BMO, NF, BBNF, and BNF‐4 are calculated to be 469, 421, 307, and 271 meV, respectively. indicating that the excitons in BNF‐4 are more feasibly dissociated into free charges by introducing dual O vacancies and interfacial bonds. The current density of the BNF heterojunction under the combined ultrasonic vibration and light conditions can be obtained by the electrochemical workstation. From Figure 4f,g and Figure S24 (Supporting Information), BNF‐4 exhibits the strongest photocurrent intensity, which is identified with the trend of piezoelectric current, indicating that the introduction of dual O vacancies and interfacial bonds could tremendously promote charge separation efficiency. On the other hand, the photo‐piezoelectric induced current density of BNF‐4 is 2.13 and 5.52 times that of under the light alone or ultrasonic vibration alone, respectively, whereas BMO, NF, and BBNF do not exhibit such a big difference. Moreover, a similar phenomenon can be observed in the Nyquist plots (Figure S25, Supporting Information), suggesting that the interfacial bonds can deplete the negative effect of the localization of polarization charges.

Above all, although vacancies can be the centers of polarization, the charge accumulation occurs mainly on the vacancies, which exacerbates and worsens the synergistic effect of photocatalysis and piezoelectricity. However, the interfacial bonds act as an “Electron Bridge” to promote charge transfer across the heterojunction, which breaks the carrier local effect and forms a positive result. Therefore, the dual built‐in electric field was formed and promotes exciton dissociation and charge transfer.

### 2.3 Piezo‐Photocatalytic Activity

**Figure 5:**
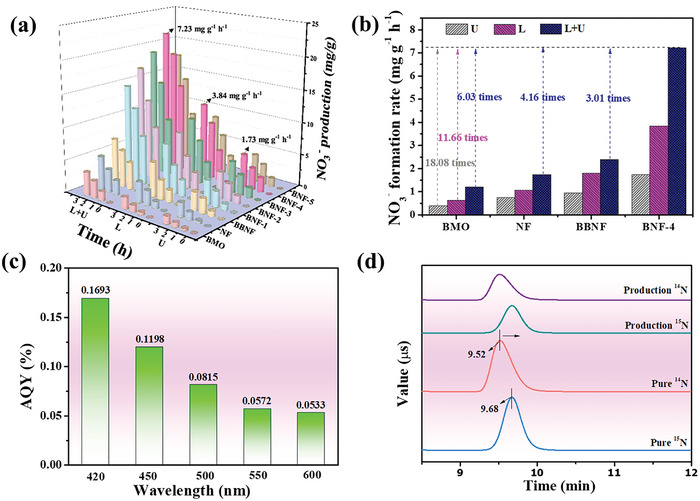
a) The NO_3_^−^ yields over as‐prepared samples. b) The NO_3_^−^ formation rates under different conditions. c) The apparent quantum efficiency of BNF‐4. d) ^15^N isotope tracing results over BNF‐4.

Nitrate is an important industrial raw material, which has been widely used in the fields of fertilizers, explosives, pharmaceuticals, dyes, and emulsifiers field.^[^ ^51^ ^]^ In this system, catalytic N_2_ oxidation with water and O_2_ experiments were also carried out. First, negligible HNO_3_ yield can be observed in the Ar atmosphere, or without using water and O_2_ catalyst, confirming the HNO_3_ are originated from N_2_ and water and O_2_. As shown in **Figure** **5**a,b and Figure S26 (Supporting Information), BNF‐4 exhibited outstanding HNO_3_ yield (3.84 mg g^−1^ h^−1^) under light irradiation, which is 6.19, 3.59, and 2.13 times that of BMO, NF and BBNF, respectively, which is assigned to BNF‐4 has excellent carrier migration performance. It is worth noting that, the quantum yield of HNO_3_ production under 420 nm can reach up to 0.17%. Moreover, BNF‐4 also displays the best HNO_3_ production rate of 7.23 mg g^−1^ h^−1^ under simultaneous light and ultrasonic irradiation. More importantly, the piezo‐photocatalytic HNO_3_ production of BNF‐4 is 1.88 and 4.18 times that of photocatalysis and piezocatalysis, respectively. which is superior to the related works. At the same time, no ammonia (NH_3_) and other by‐product were detected in ion chromatography and ^1^H NMR and gas chromatography (Figure S27, Supporting Information), further indicating the high selectivity of BNF‐4. To study the source of N_2_, the ^15^N_2_ isotopic labeling experiment was conducted. A typical ^15^NO_3_^−^ signal can be observed, suggesting that the nitrate is derived from the PPNN. Lastly, the catalytic cycles have been carried out under different condition. As shown in Figures S28–S31 (Supporting Information), there is no noticeable decrease in HNO_3_ generation rates over BNF‐4 after ten catalytic cycles. There are also no significant changes in chemical composition, suggesting the high stability of BNF‐4. Above all, BNF‐4 is the ideal catalyst for the catalytic oxidatively fixing of N_2_ with O_2_ and water into nitrate.

### 2.4 . Nitrogen Oxidation Mechanism

**Figure 6:**
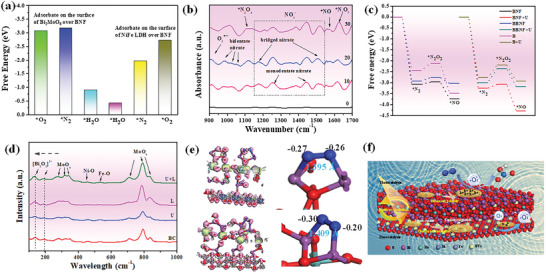
a) Adsorption energies of reactant molecule over the different site of BNF‐4. b) In situ FT‐IR spectra for PNN over BNF‐4. c) Gibbs free energy for PPNN over the prepared samples. d) In situ Raman spectrum of the prepared samples, e) differential charge and charge distribution of BNF. f) Proposed process for PPNN over BNF‐4.

First, the surface properties play important roles in nitrogen oxidation reaction. As exhibited in Figure S32 (Supporting Information), the water contact angles of BMO, NF, BNF, and BBNF are 46.2°, 25.8°, 24.2°, and 18.2°, suggesting that the interfacial bond adjusts the inner structure and results in more hydrophilic character of BNF, which contributes to the subsequent activation of *NO*_x_*. We further tested the surface activated capacity of O_2_ and N_2_ by TPD measurement (Figure S33, Supporting Information). Reasonably, a broad O_2_ peak in the 60–180 °C appeared, and a weak N_2_ peak in the range of 60–180 °C appeared, proving the oxygen is preferentially activated over the as‐prepared samples. Moreover, the highest concentration of N_2_ and O_2_ over BNF demonstrates that the O vacancies and interfacial bonds distribution could provide more active sites for PPNN. In addition, the DFT calculation has been carried out to investigate the active site. As shown in **Figure** **6**a and Figure S34 (Supporting Information), the oxygen vacancy in NF preferentially activates the O_2_, which agrees with the experimental data. Those results also suggested that the interfacial bond regulates the electronic interaction of BNF and results in lots of electrons gathering in NF for activating oxygen.

Based on the above analysis, the free radical quenching experiments were carried out to explore the underlying principle. As shown in Figure S35 (Supporting Information), the superoxide radical and holes play a major role to participate in the oxidation of N_2_. To further explore the real routes of catalytic oxidative fixation of N_2_ with O_2_ and water into nitrate, in situ DRIFTS spectra were performed. As exhibited in Figure 6b, the peaks at 930, 1125, and 1623, 1558 cm^−1^ are the characteristic peaks of O_2_^.−^, *N_2_O_2_ and *NO,^[^ ^3^, ^52^ ^]^ respectively. In addition, other peaks can be vested in the different adsorption states of NO_3_^−^.^[^ ^3^ ^]^ To investigate the mechanism for oxidatively fixing N_2_ into nitrate on the molecular level, DFT calculations have been conducted. As shown in Figure 6c, the determining step is *N_2_→*N_2_O_2_. In summary, the nitrogen fixation process can be determined by the following steps: 1) electrons participate in the formation of O_2_^.−^; 2) the holes, O_2_^.−^ and H^+^ are involved in the activation of N_2_ to form NO; 3) the synergistic effect of NO, hole and H_2_O drives the direct synthesis of NO_3_^−^. Importantly, the mechanical stress greatly decreases the Gibbs free energy of the nitrogen oxidation, suggesting the mechanical stress promotes the activation of reactants (Figure 6c; Figures S36 and S37, Supporting Information).

Due to the variable internal structure, further manipulation of the reaction process over BNF‐4 is possible through piezoelectricity. As shown in Figure 5d, the in situ Raman spectrum, the vibration peaks generated by Bi‐Bi A_lg_ mode (94 cm^−1^) and Bi‐O (382 cm^−1^) are redshifted under light and pressure,^[^ ^32^, ^53^ ^]^ proving that the stretching of the bond length leads to internal reconstruction, which is identified with the DFT results. On the other hand, the charge difference and bond length difference between two adsorbed N≡N bonds on the BNF are 0.01e and 1.395Å, respectively. which are larger than those of BBNF (Figure S38, Supporting Information), suggesting that the interfacial bonds regulated electronic interaction and promote the local polarization of N_2_, which accelerates the fracture of the N≡N bonds. Moreover, the local polarization and fracture of N≡N bonds are more obvious over BNF under 100 MPa, suggesting that the electronic interaction of the interfacial bonds induces internal reconstruction more rapidly under ultrasound, which reinforces the reaction process. In addition, the mechanical stress greatly decreases the Gibbs free energy of the determining step of N_2_→*N_2_O_2_, which reduces the reaction resistance, suggesting that the piezoelectricity not only provides a huge electric field to promote charge transfer, but also induces internal reconstruction to reduce the activation energy of the reaction. Based on the above analysis, the mechanism has been proposed (Figure 6f). Under visible light irradiation and ultrasonic vibration, the electrons and holes are generated. With the assistance of internal electric field and interfacial bonds, the e^−^ on CB of BMO and h^+^ on the VB of NF can recombine together. While the remaining h^+^ in the VB of BMO, e^−^ is migrates into the O vacancies and reacts with O_2_ to form superoxide radical. The superoxide radical can attack the N_2_ to generate NO, and finally form HNO_3_.

## 3 Conclusion

In summary, the oxygen defect reached NiFe‐layered double hydroxide‐Bi_2_MoO_6−_ *_x_* heterojunctions (BNF) was successfully constructed via facile hydrothermal method. The optimized BNF‐4 displayed an excellent HNO_3_ production rate (7.23 mg g^−1^ h^−1^) as well as good recycling stability under visible light irradiation and ultrasonic vibration. Sufficient evidence revealed that the dual oxygen vacancies in the NiFe‐layered double hydroxide and Bi_2_MoO_6−_ *_x_* induced interfacial bonds, which acted as “charge bridge” and “strain center” to break the carrier local effect and form a positive correlation with piezocatalysis and photocatalysis. Therefore, the dual built‐in electric field was formed and promoted exciton dissociation and charge transfer. On the other hand, the strong electronic interaction in the interfacial bond led to internal reconstruction for promoting the local polarization and adsorption of N_2_, which accelerated the fracture of the N≡N bonds and reduced the activation energy of the reaction. Our work paves a new horizon for the relationship of the ingenious construction of internal structure for regulating charge migration path in catalyst, as well as material design encompasses the fabrication of heterojunctions or the regulation of surface/interface properties.

## 4 Experimental Section

### Preparation of Bi_2_MoO_6_ (BMO) Nanosheet

Generally, 0.242 g of Na_2_MoO_4_·2H_2_O, 0.3 g of cetyltrimethylammonium bromide (CTAB) and 0.97 g of Bi(NO_3_)_3_·5H_2_O were mixed in 80 mL of H_2_O under stirring for 30 min. Then, the above solution was added into a 100 mL Teflon‐lined autoclave at 180 °C for 16 h. Finally, the BMO was obtained by following the operation containing washing, filtration, and drying.

### Preparation of NiFe Hydrotalcite (NF) Nanosheet

In a typical procedure, 1.783 g of NiCl_2_·6H_2_O and 1.010 g of Fe(NO_3_)_3_·9H_2_O were dispersed in 100 mL of deionized water. Then, 20 mL of formamide solution with a volume fraction of 23% and 10 mL of 2.5 mol NaOH were added into 20 mL of the above solution. Then, the solution was reacted in an oil bath with pH 10 and constant temperature of 80 °C for 10 min. Finally, the monolayer NF was collected by a series of washing, filtration, and drying.

### Preparation of Bi_2_MoO_6_ and NiFe Hydrotalcite (BNF) Heterojunction

Typically, 1.0 g of the prepared Bi_2_MoO_6_ and 0.1 g of NiFe hydrotalcite were dispersed in 60 mL of deionized water under stirring for 12 h. After 1 h of ultrasound, the above solution was transferred to a 100 mL Teflon‐lined autoclave and heated at 120 °C for 12 h. Finally, the product was collected by a series of washing, filtration, and drying, which can be donated as BNF‐1. In addition, the different mass ratios of Bi_2_MoO_6_ and NiFe hydrotalcite were prepared by the same methods except adding the different amounts of the NiFe hydrotalcite (0.2, 0.3, 0.4, and 0.5 g), which donated as BNF‐2, 3, 4, and 5.

### Preparation of Bulk Randomly Compounded Bi_2_MoO_6_ and NiFe Hydrotalcite (BNF) Heterojunction (BBNF)

Typically, 1.0 g of the prepared Bi_2_MoO_6_ and 0.4 g of NiFe hydrotalcite were dispersed in 60 mL of deionized water under stirring for 12 h. Then, the product was collected by a series of washing, filtration, and drying.

## Conflict of Interest

The authors declare no conflict of interest.

## Supporting information

Supporting Information

## Data Availability

The data that support the findings of this study are available from the corresponding author upon reasonable request.
