# Supplementary material for: Dynamic Reconfiguration and Local Polarization of NiFe‐Layered Double Hydroxide‐Bi2MoO6− x Heterojunction for Enhancing Piezo‐Photocatalytic Nitrogen Oxidation to Nitric Acid
Source: Adv Sci (Weinh). 2024 Apr 16;11(25):2401667. doi: 10.1002/advs.202401667 (PMC11220699; doi:10.1002/advs.202401667)
Supplement: Supplementary file 1 — Supporting Information [file ADVS-11-2401667-s001.pdf]

ADVANCED SCIENCE
Open Access

Supporting Information

for *Adv. Sci.*, DOI 10.1002/advs.202401667

Dynamic Reconfiguration and Local Polarization of NiFe-Layered Double Hydroxide-Bi_2MoO_{6-x} Heterojunction for Enhancing Piezo-Photocatalytic Nitrogen Oxidation to Nitric Acid

*Xiaoxu Deng, Peng Chen, Ruirui Cui, Xingyong Gong, Yubo Wu, Xu Wang and Chaoyong Deng**

Supporting Information

Dynamic Reconfiguration and Local Polarization of NiFe-Layered Double Hydroxide-Bi_2MoO_{6-x} Heterojunction for Enhancing Piezo-photocatalytic Nitrogen Oxidation to Nitric acid

Xiaoxu Deng, Peng Chen, Ruirui Cui, Xingyong Gong, Yubo Wu, Xu Wang and Chaoyong Deng[*]

X. Deng, Dr. C. Cui, Dr. X. Gong, Y. Wu, Prof. X. Wang,
Key Laboratory of Electronic Composites of Guizhou Province, College of Big Data and Information Engineering, Guizhou University, Guiyang, 550025, Guizhou, China

Prof. C. Deng
Key Laboratory of Electronic Composites Materials of Guizhou Province, College of Big Data and Information Engineering, Guizhou University, Guiyang, 550025, Guizhou, China

School of Electronics and Information Engineering, Guiyang University, Guiyang 550005, Guizhou, China
E-mail: cydeng@gzu.edu.cn (C. Deng)

Dr. P. Chen
Key Laboratory of Green Chemical and Clean Energy Technology of Guizhou Provincial, School of Chemistry and Chemical Engineering, Guizhou University, Guiyang 550025, Guizhou, China

Experimental Section

Characterization

The X-ray diffraction (XRD, SmartLab), Raman spectra and X-ray photoelectron spectroscopy (XPS, ESCALAB 250Xi, Thermo) were selected to explore the detailed crystalline structure and surface electronic states of as prepared samples, respectively. In addition, electron paramagnetic resonance (EPR) spectra were carried out by the Bruker EMXplus. The morphology of as prepared samples was employed by scanning

electron microscopy (SEM, Hitachi SU8100, 5 kV, Japan) and transmission electron microscopy (TEM). The light absorption of the prepared samples was recorded by the Diffuse reflection spectra (Shimadzu U-3600 spectrophotometer). The photoluminescence (PL) spectra were investigated on a FLS 980 luminescence spectrometer. In situ FTIR spectra of BNF-4 were tested by Thermo Fisher. The photocurrent and Mott-Schottky plots were evaluated on CHI 660E electrochemical workstation with three-electrode.

Activity evaluation

The piezo-photocatalytic performance of NOR were performed in a glass vessel under visible-light irradiation by a 300 W Xe lamp and ultrasonic condition. In a typical experiment, 20 mg catalyst was dispersed in 50 mL of deionized water. Before reaction, high-purity N_2 and O_2 was bubbled into the above solution and stirred for 30 min. Under appropriate irradiation and ultrasonic time, 5 mL of the solution was experienced centrifugation and detected by ion chromatograph (ICS-900).

Computational methods

The density functional theory (DFT) simulations are implemented in Material studio software with CASTEP modes. A vacuum thickness and residual atomic forces were set as 20 Å and 0.05 eV Å. The Monkhorst−Pack k-points was used for 3 × 3 × 1, and the energy cutoff was selected as 450 eV. The adsorption energy of adsorbates was calculated according to the reported works.

The anomalous piezoelectricity of BNF was explored by COMSOL

Multiphysics® software. In our model, the piezoelectric interface consists of a "Structural Mechanics Module" and an "Electrostatics Interface" coupled together by the multiphysical properties of the piezoelectric effect.

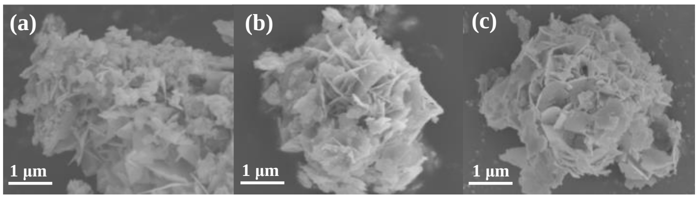

Figure S1. SEM images of (a) BMO, (b) NF and (c) BBNF.

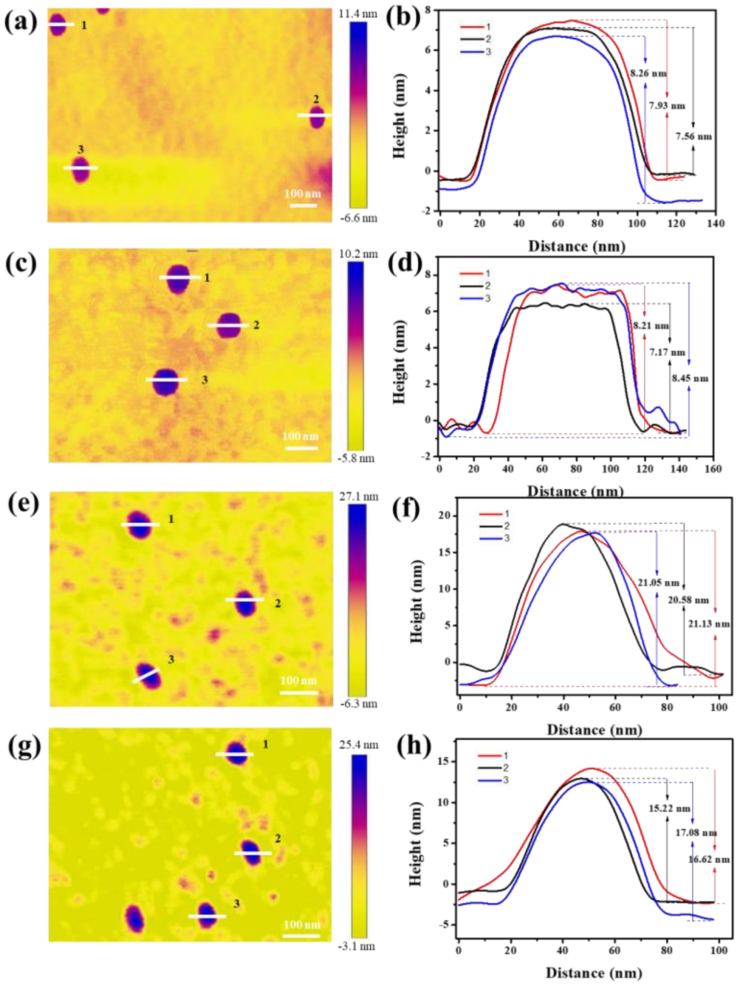

Figure S2. AFM images and the corresponding height profiles of (a,b) BMO, (c,d) NF, (e,f) BBNF and (g,h) BNF-4.

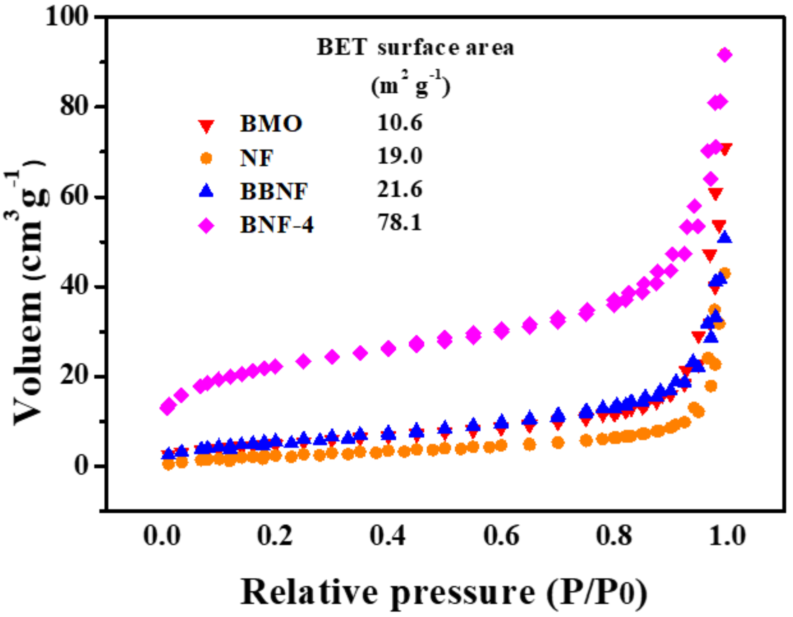

Figure S3. N_2 sorption isotherms of as-fabricated with the BET surface area data provided as inset.

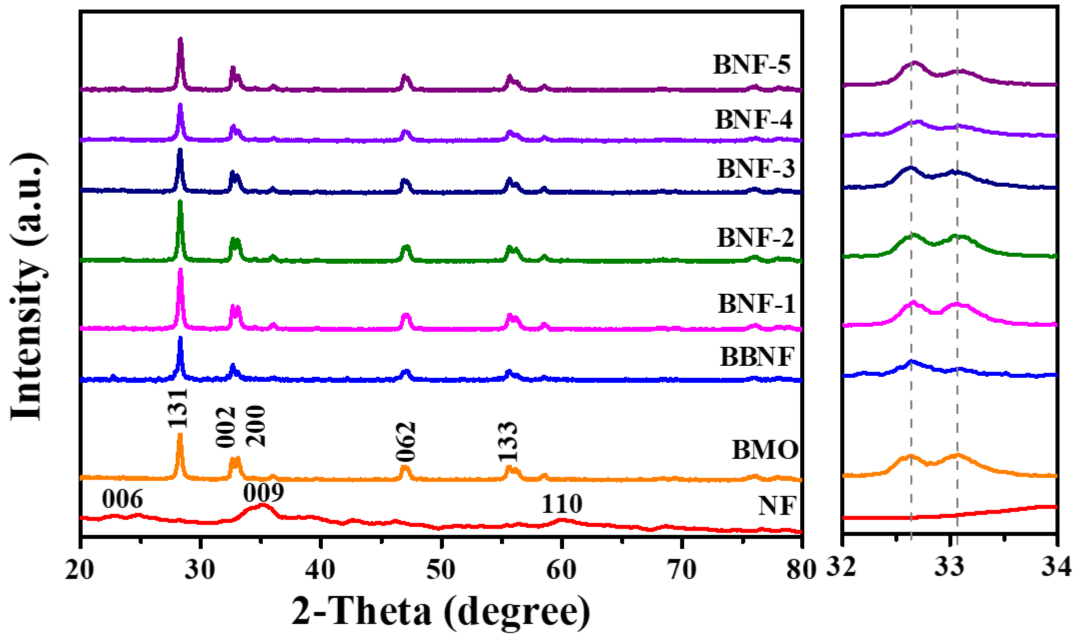

Figure S4. XRD spectra of as-obtained samples.

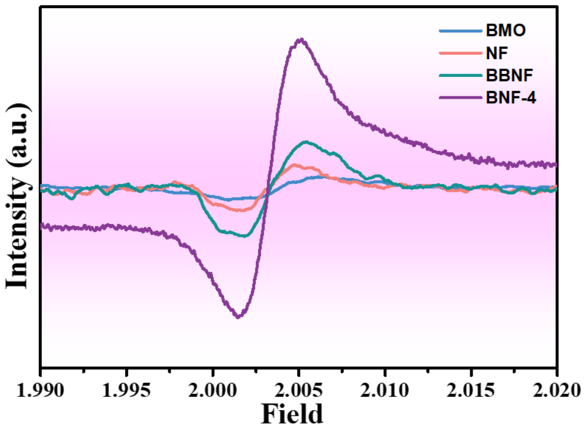

Figure S5. EPR spectra of the oxygen vacancy in BMO, NF, BBNF, BNF-4.

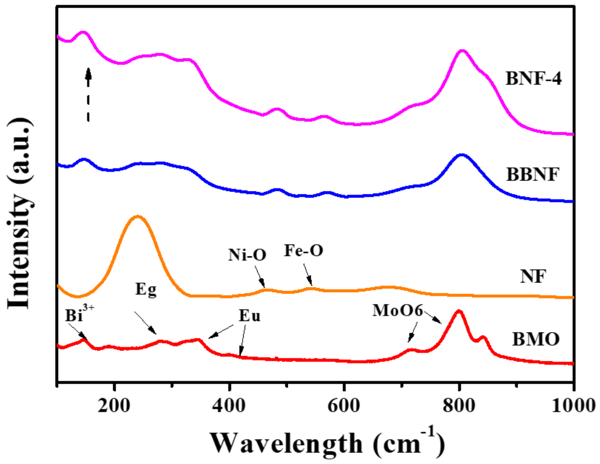

Figure S6. Raman spectra of as-obtained samples.

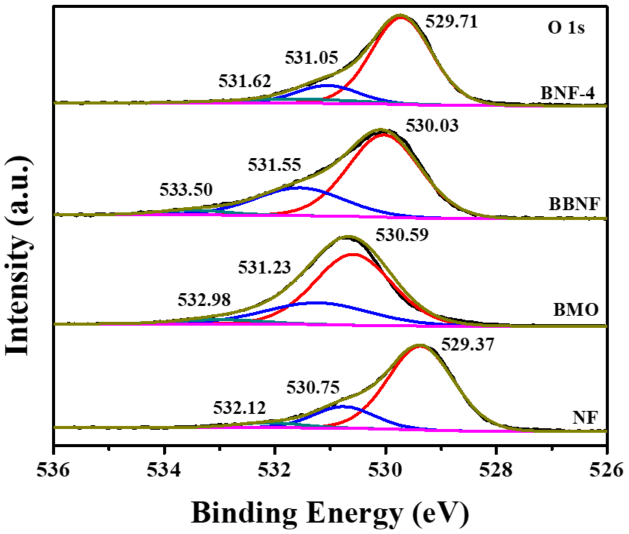

Figure S7. XPS spectra of O 1s for prepared samples.

As shown in Figure S7, the deconvoluted O 1s spectra of BNF-4 at 529.71,

531.05 and 531.62 eV are coordinated to the presence of metal-oxygen bonds, defective oxygen and adsorbed oxygen. Moreover, the deconvoluted O 1s spectra of BMO at 530.59, 531.23 and 532.98 eV are belonged to the presence of metal-oxygen bonds, defective oxygen and adsorbed oxygen. There peaks at 529.37, 530.75 and 532.12 eV are related to the presence of metal-oxygen bonds, defective oxygen and adsorbed oxygen in NF. Compared with the BMO and NF, all the O 1s spectra of BNF-4 are harmony with the trends in other elements, indicating the electron transfer from the BMO to the NF, and the strong electronic interaction in the interfacial bond.

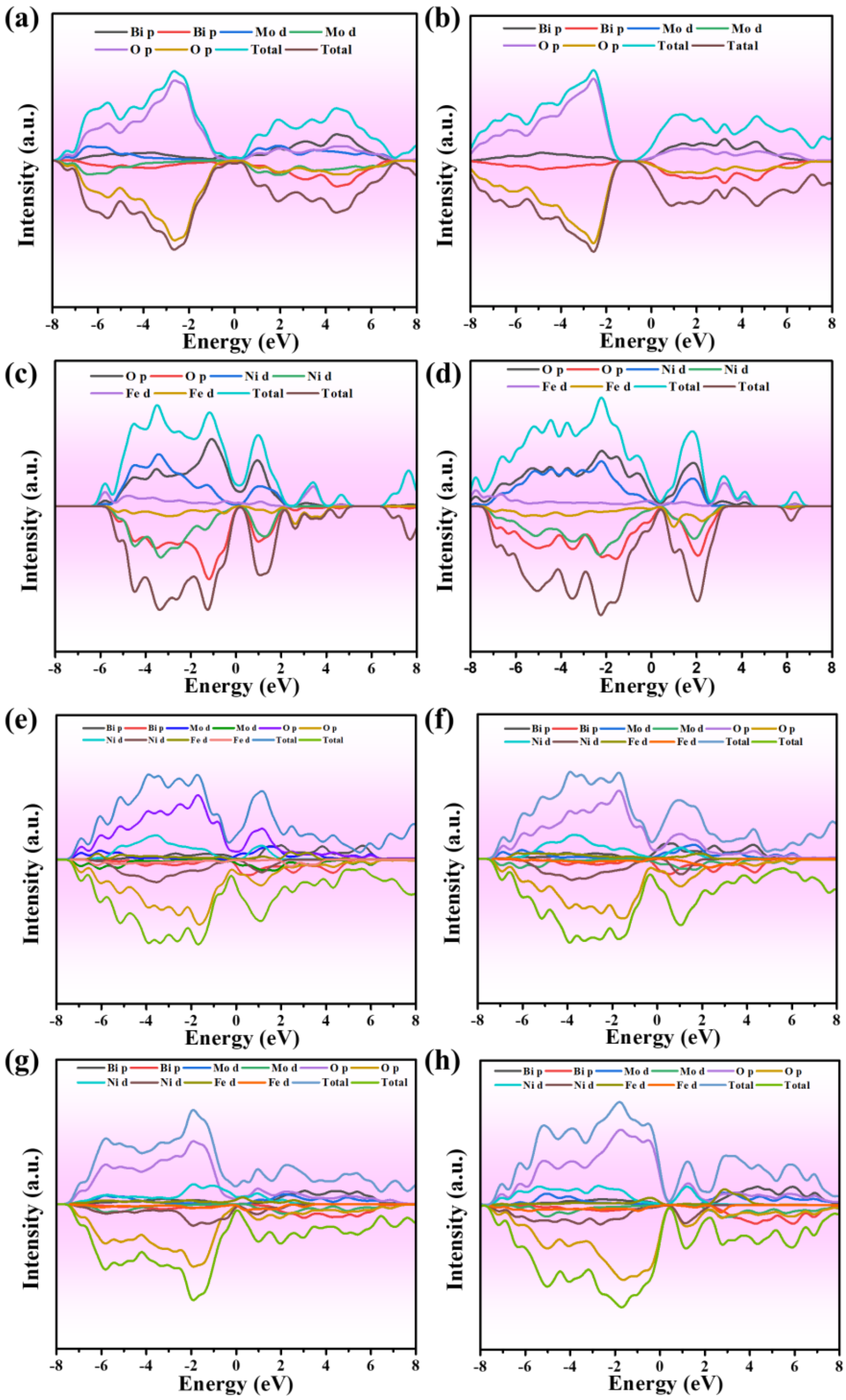

Figure S8. DFT-calculated density of states (DOS) of (a) BMO under no pressure, (b) BMO under 100 MPa pressure. (c) NF under no pressure, (d) NF under 100 MPa pressure. (e) BBNF under no pressure, (f) BBNF under 100 MPa pressure. (g)BNF-4 under no pressure and (h) BNF-4 under 100 MPa pressure.

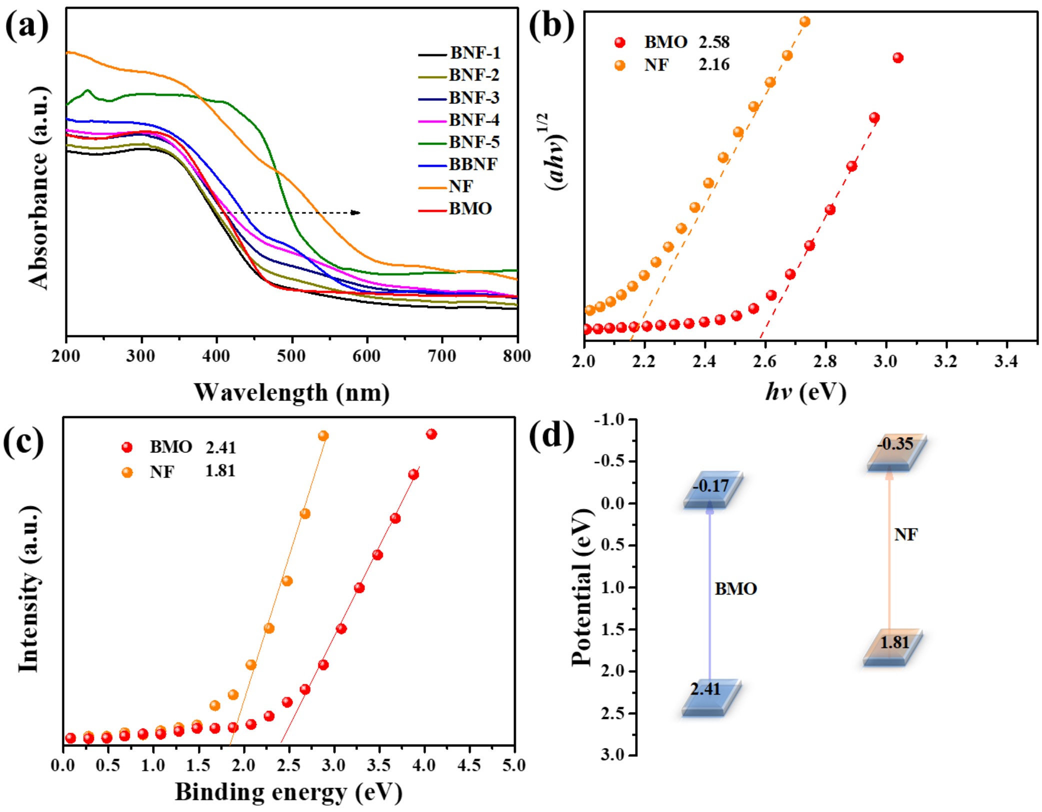

Figure S9. (a) UV–Vis DRS spectra, (b) Tauc plots, (c) XPS valence band spectra and (d) schematic diagram of electronic band structure of as prepared samples.

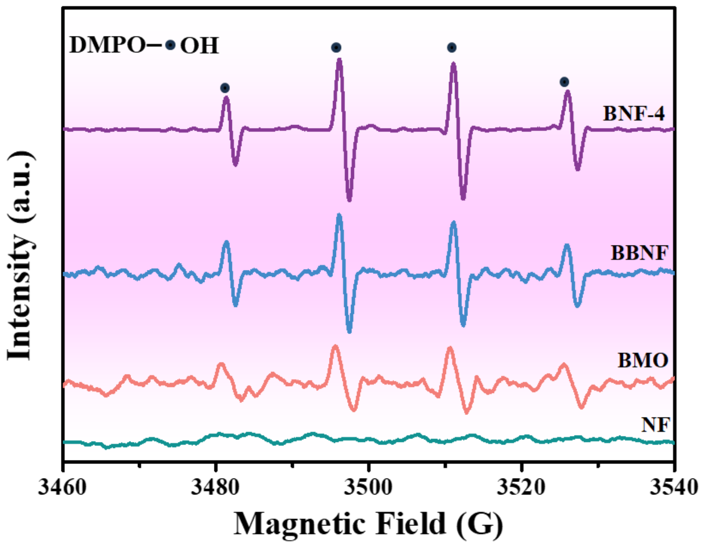

Figure S10. EPR spectra of hydroxyl groups in NF, BMO, BBNF, BNF-4.

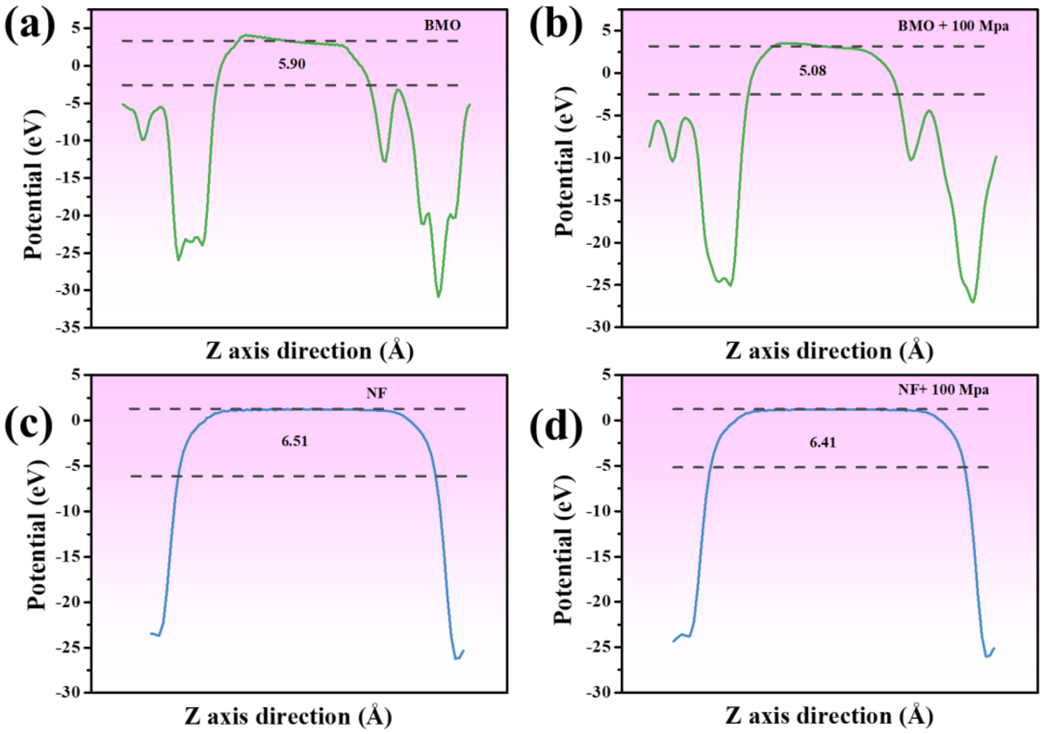

Figure S11. The work functions of (a) BMO under no pressure, (b) BMO under 100 MPa pressure, (c) NF under no pressure and (d) NF under 100 MPa pressure.

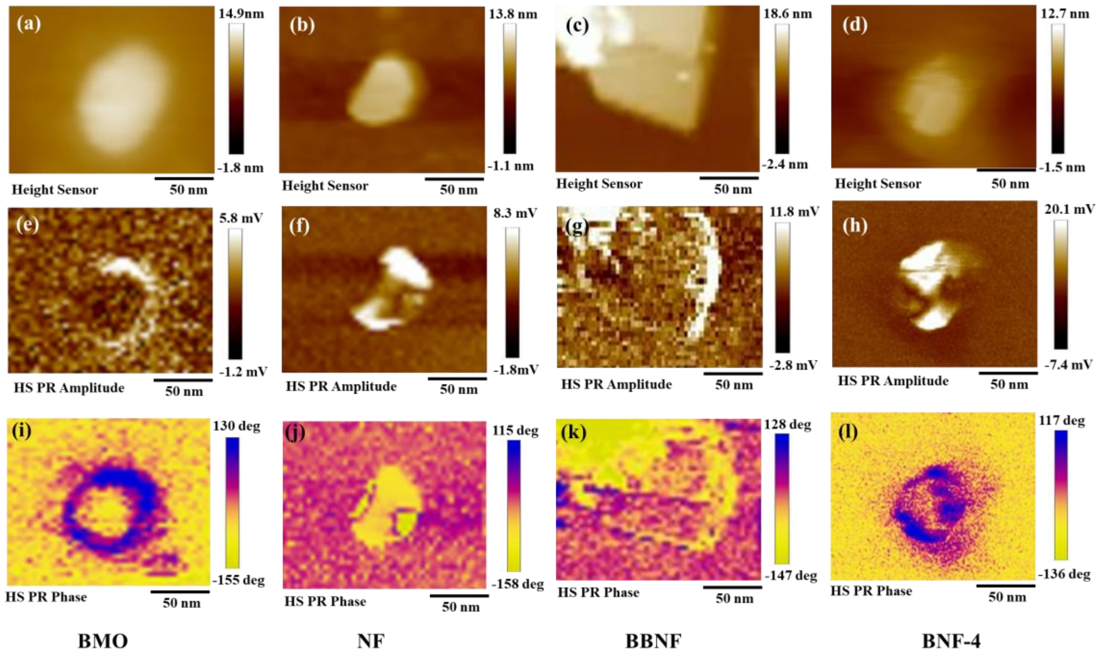

Figure S12. PFM image for BMO: (a) Topography image, (e) amplitude image (i) phase image; NF: (b) Topography image, (f) amplitude image (j) phase image; BBNF:

(c) Topography image, (g) amplitude image (k) phase image; BNF-4: (d) Topography image, (h) amplitude image (l) phase image.

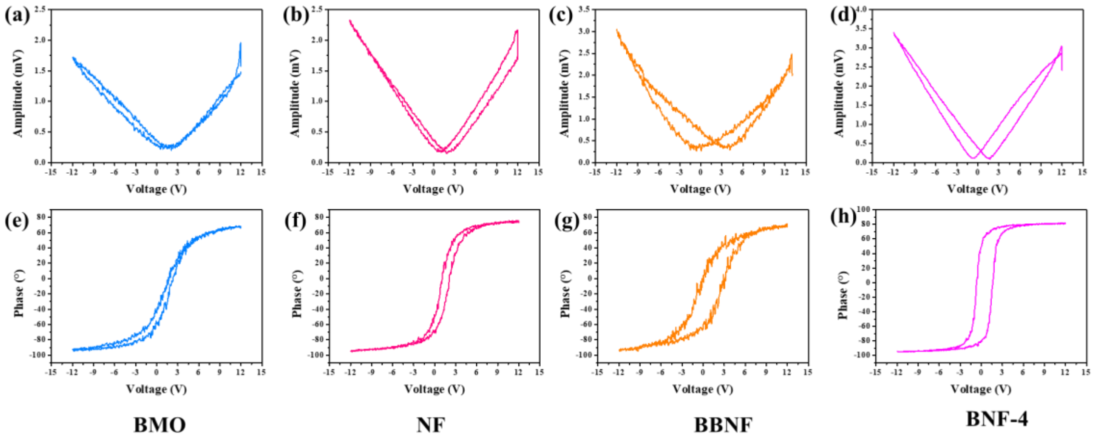

Figure S13. Piezoresponse amplitude butterfly loops of BMO(a), NF(b), BBNF(c) and BNF-4(d); phase hysteresis loops of BMO(e), NF(f), BBNF(g) and BNF-4(h).

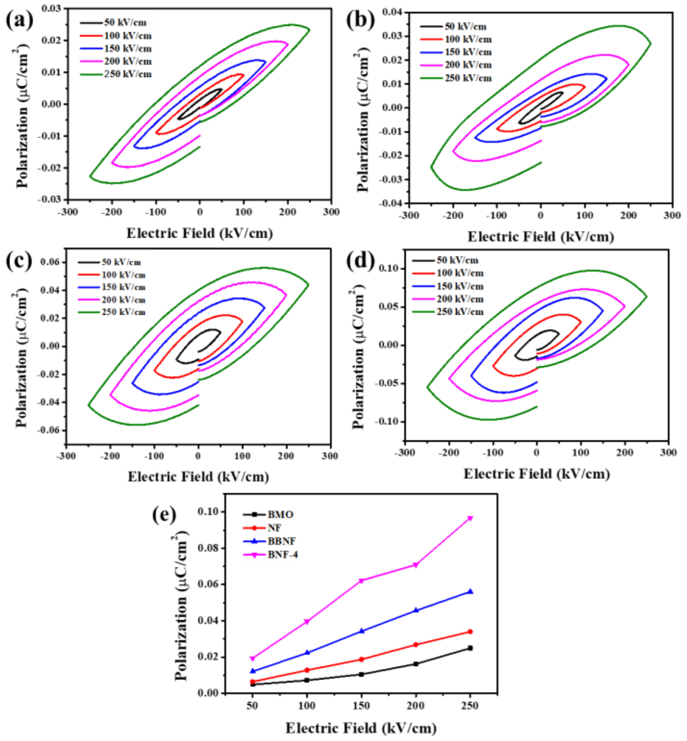

Figure S14. P–E loops of (a) BMO, (b) NF, (c) BBNF, (d) BNF-4 (e) Polarization the prepared samples.

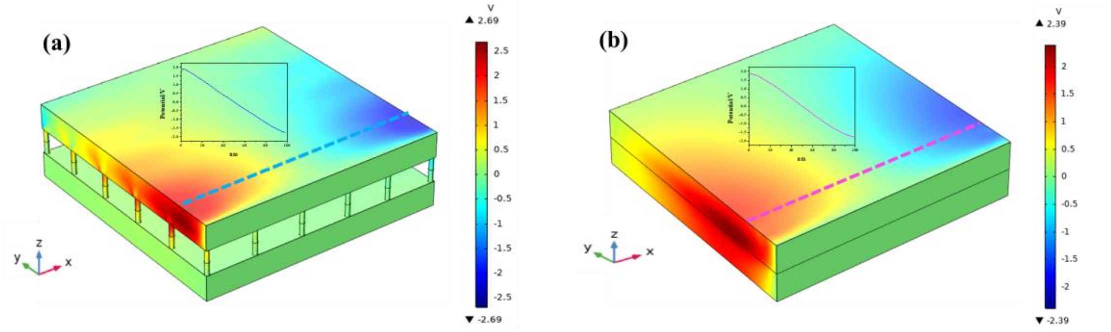

Figure S15. COMSOL simulate calculation of piezoelectric potential for (a) BNF-4, (b) BBNF.

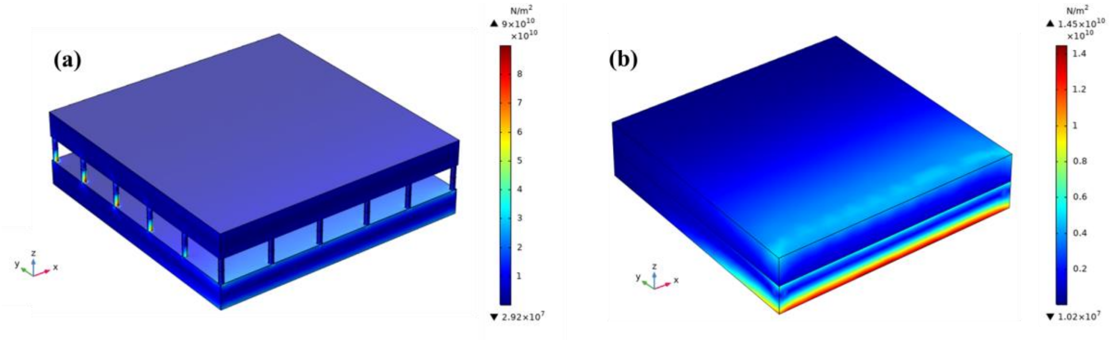

Figure S16. COMSOL simulate calculation of stress distribution for (a) BNF-4, (b) BBNF.

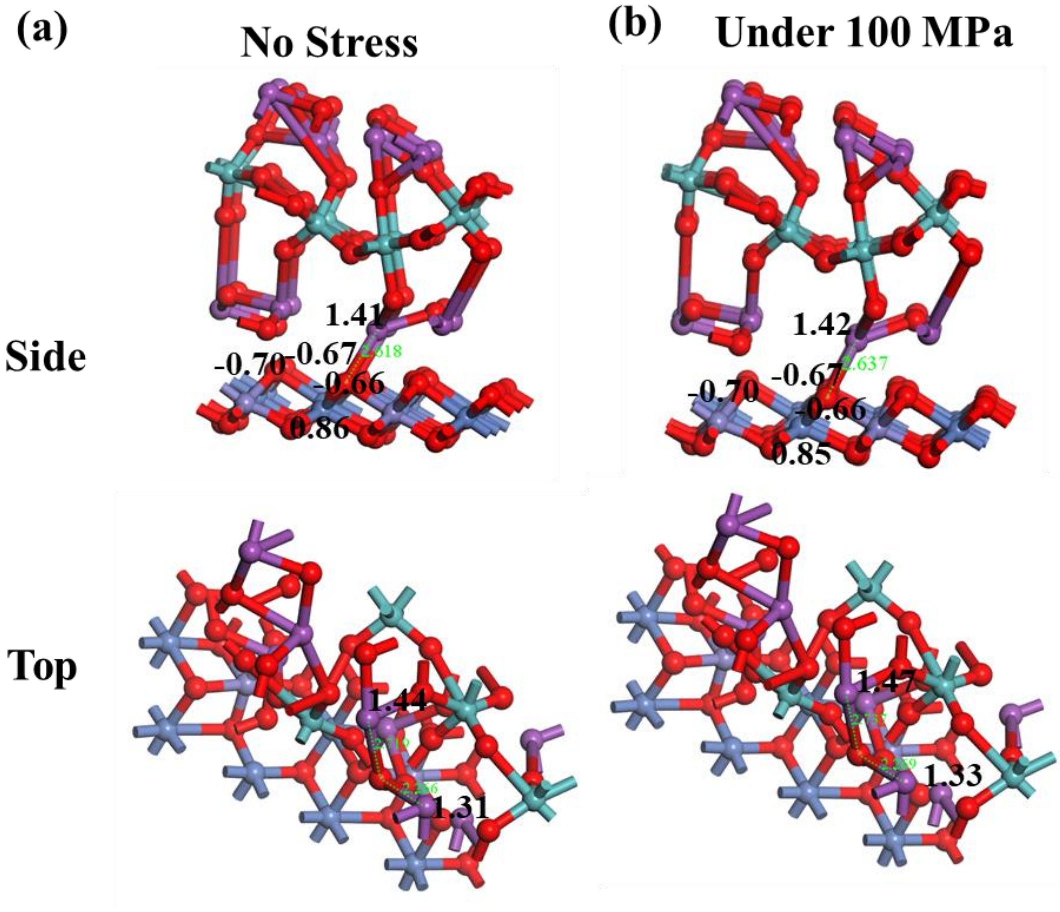

Figure S17. The charge distribution of BNF-4 under (a) No stress, (b)100 MPa.

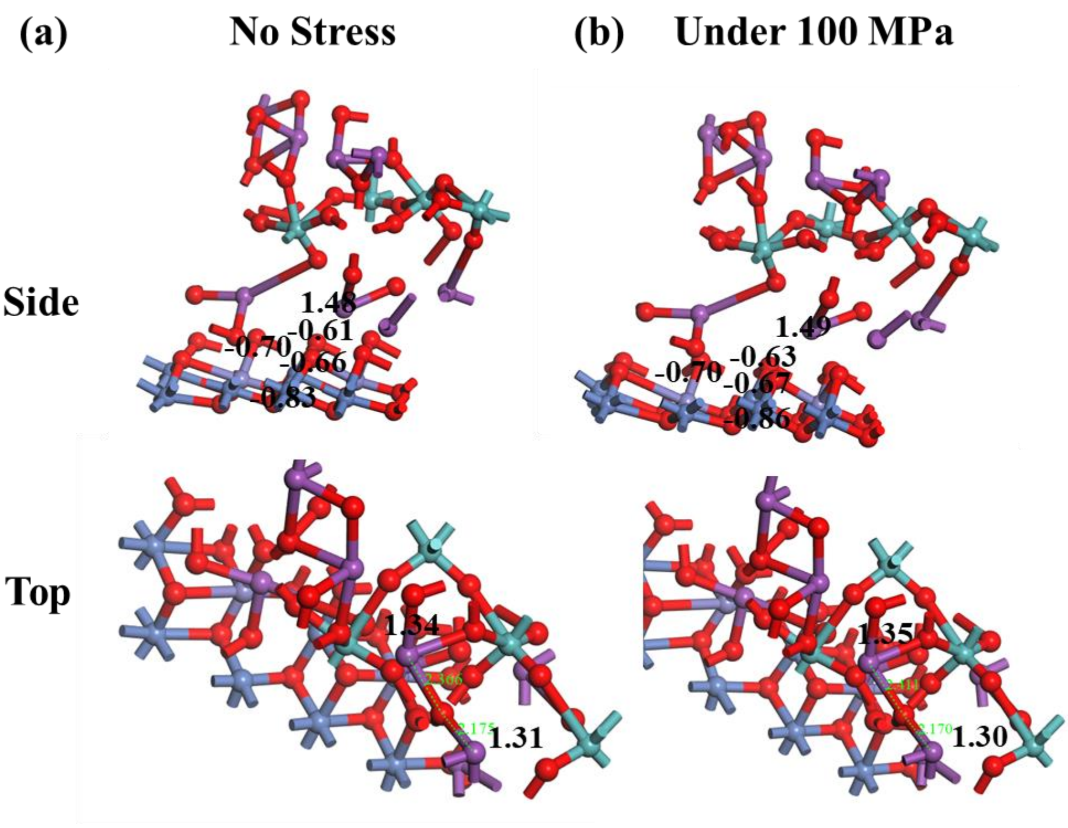

Figure S18. The charge distribution of BBNF under (a) No stress, (b)100 MPa.

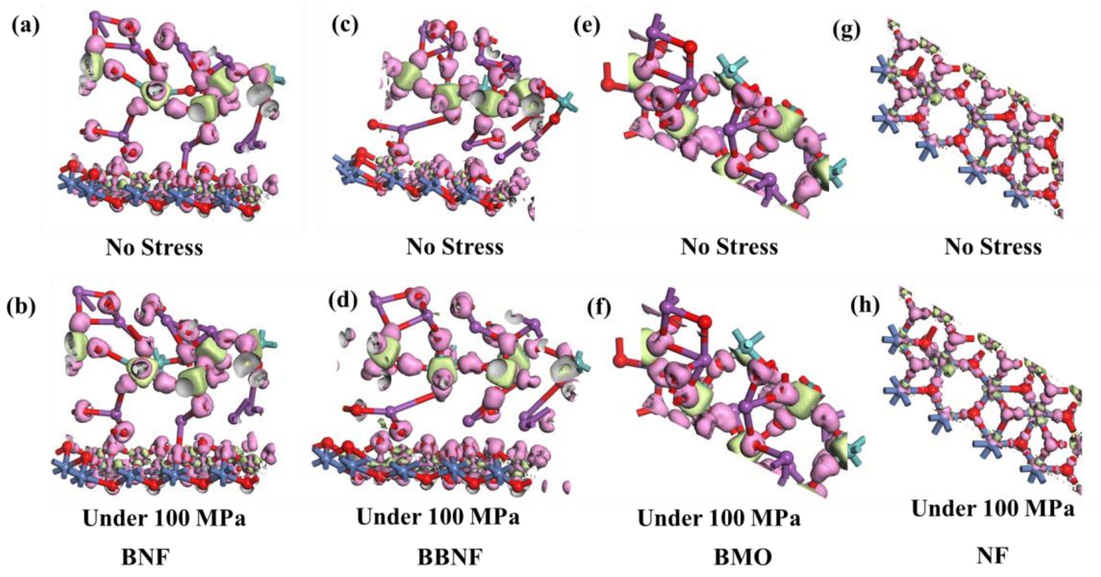

Figure S19. Charge difference distribution of BNF under (a) No stress, (b)100 MPa. BBNF under (c) No stress, (d)100 MPa. BMO under (e) No stress, (f)100 MPa. NF under (g) No stress, (h)100 MPa.

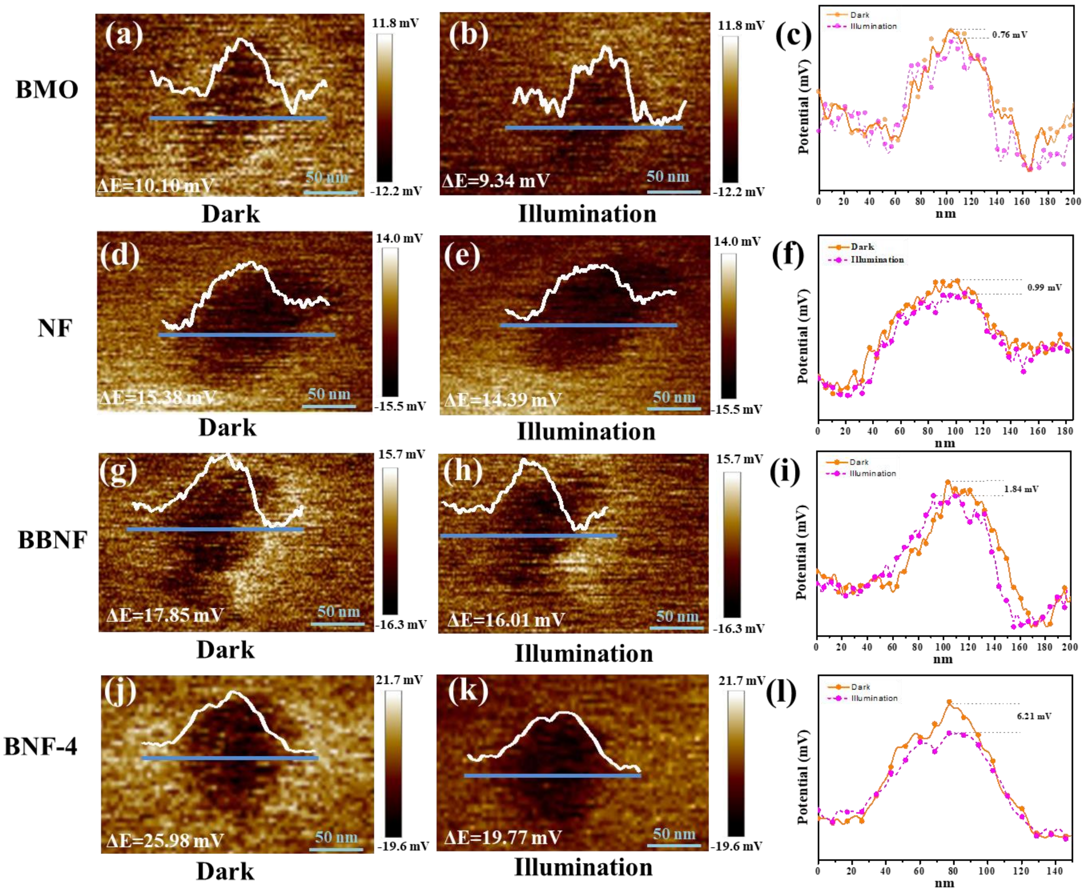

Figure S20. KPFM of (a-c) BMO, (d-f) NF, (g-i) BBNF and (j-l) BNF-4 in the dark or under illumination.

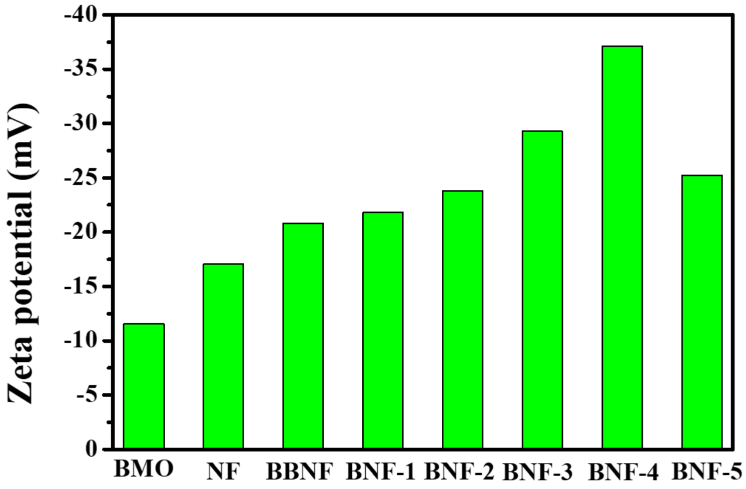

Figure S21. Zeta potential of as prepared sample.

Table S1. Zeta potentials of the as-prepared photocatalysts.

Test time	1	2	3	4	5	Average
**BMO**	-12.659	-11.156	-12.341	-10.574	-11.091	-11.564
**NF**	-16.152	-18.406	-15.331	-19.217	-16.188	-17.058
**BBNF**	-21.497	-21.630	-19.209	-22.555	-19.271	-20.832
**BNF-1**	-19.882	-23.419	-22.642	-20.392	-22.656	-21.798
**BNF-2**	-25.757	-23.899	-22.932	-22.549	-23.821	-23.792
**BNF-3**	-28.286	-30.102	-28.759	-29.288	-30.025	-29.292
**BNF-4**	-38.001	-35.327	-37.313	-36.564	-38.375	-37.116
**BNF-5**	-23.937	-23.901	-27.520	-24.315	-26.453	-25.225

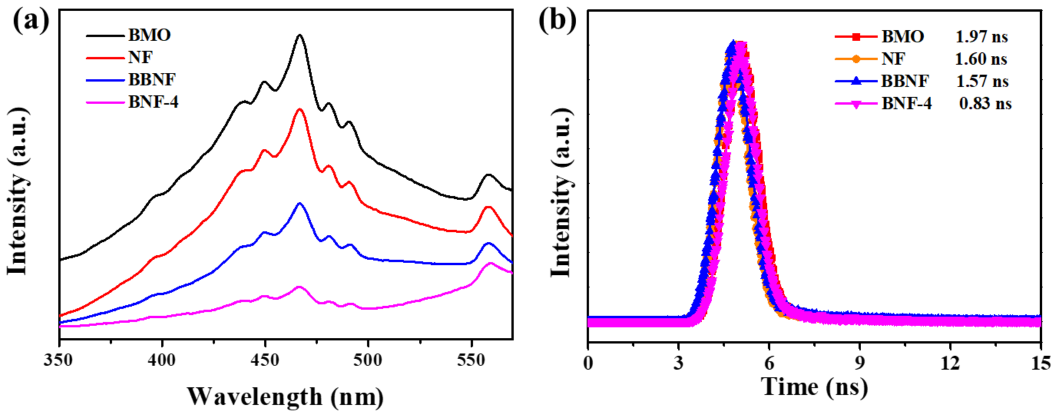

Figure S22. (a) The PL spectra of as-obtained samples, (b) Time-resolved transient PL decay (TRPL) spectra of as-obtained samples.

Table S2. The values of TRPL lifetime τ_i and corresponding constant A_i (λ=450 nm)

Sample	A_1 (%)	τ_1 (ns)	A_2 (%)	τ_2 (ns)	A_3 (%)	T_3 (ns)	τ_{ave} (ns)
**BWO**	91.27	0.1205	5.81	0.6379	2.93	4.0851	1.97
**NF**	95.11	0.1426	4.89	2.9668			1.60
**BBNF**	85.48	0.1070	9.86	0.6109	4.66	2.9655	1.57
**BNF-4**	95.62	0.1483	4.38	1.9583			0.83

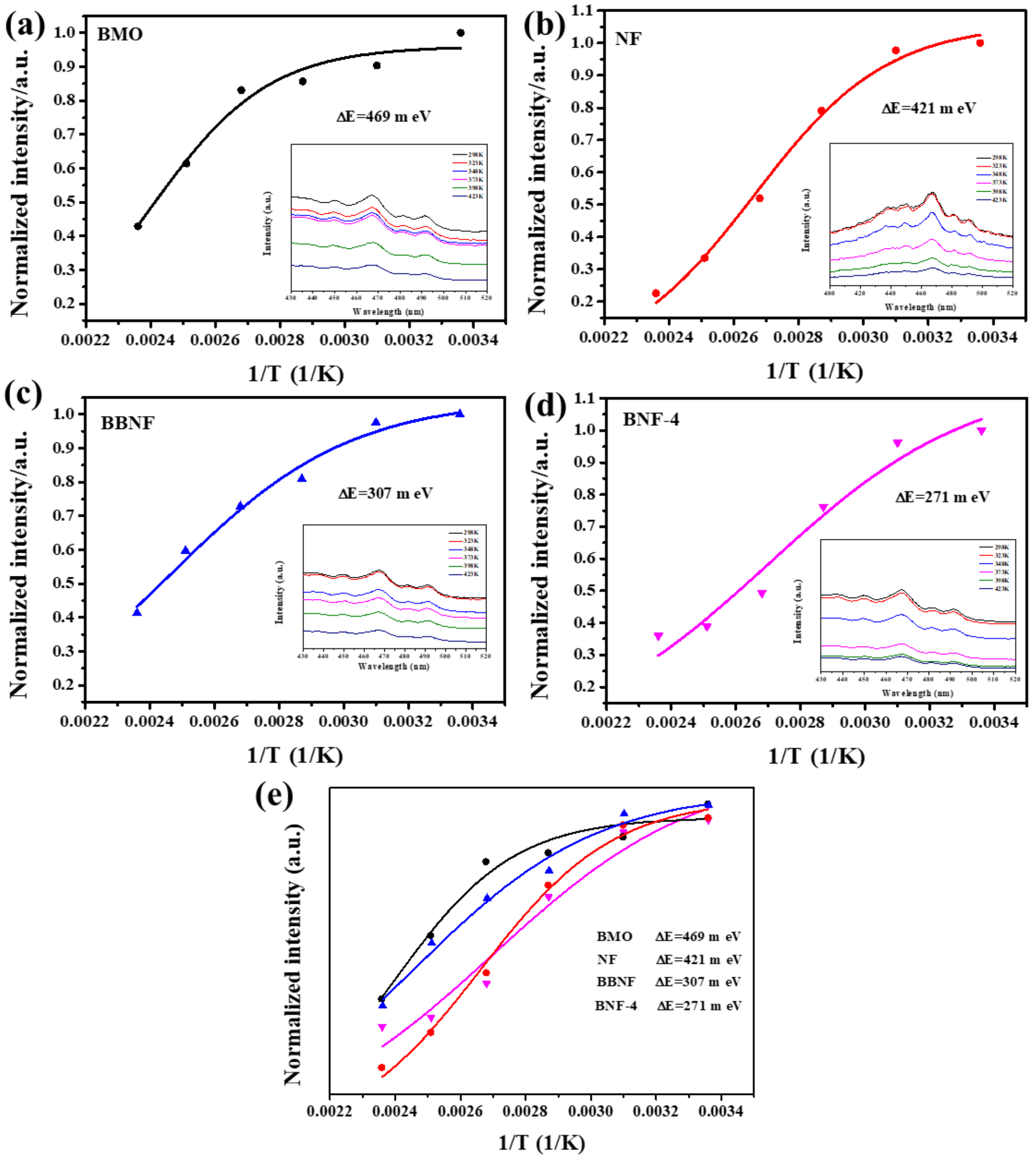

Figure S23. TD-PL spectra of (a) BMO, (b) NF, (b) BBNF and (d) BNF-4 under 330 nm laser excitation.

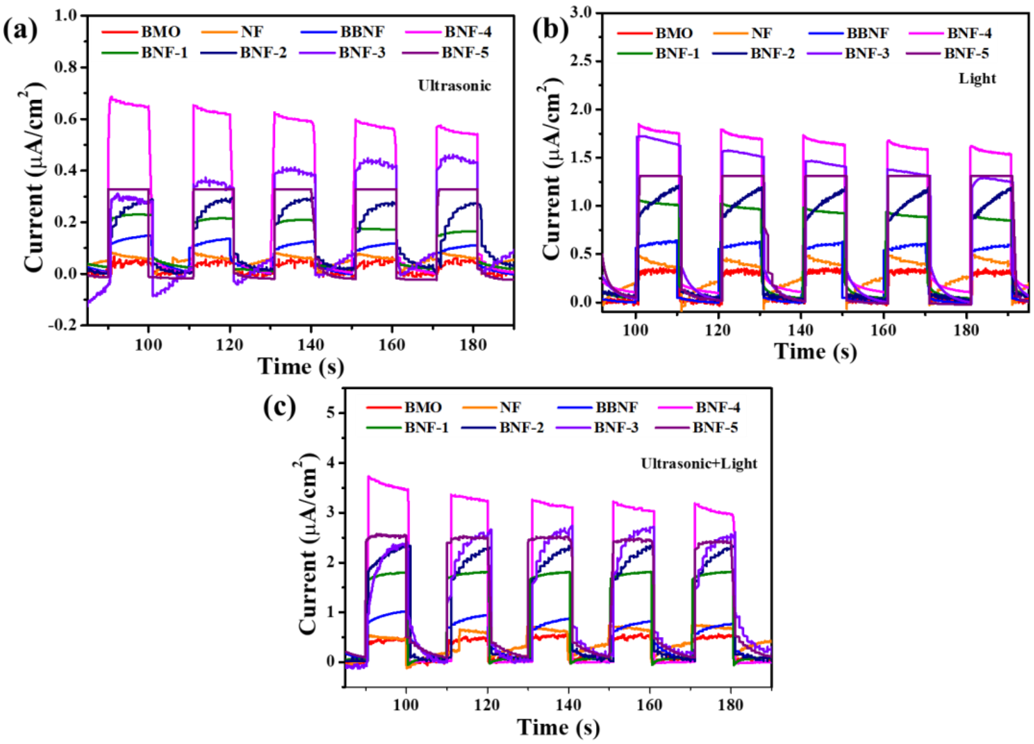

Figure S24. Transient current responses of as prepared samples (a) under ultrasonic, (b) light, (c) under light and ultrasonic conditions.

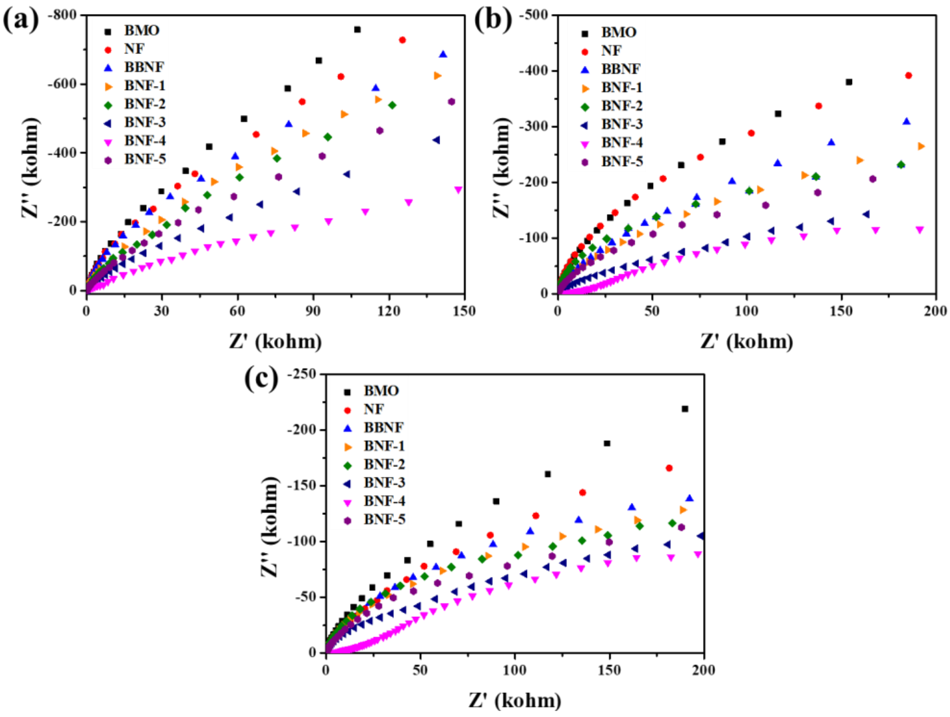

Figure S25. EIS Nyquist polts of as prepared samples (a) under ultrasonic, (b) under light, (c) under light and ultrasonic conditions.

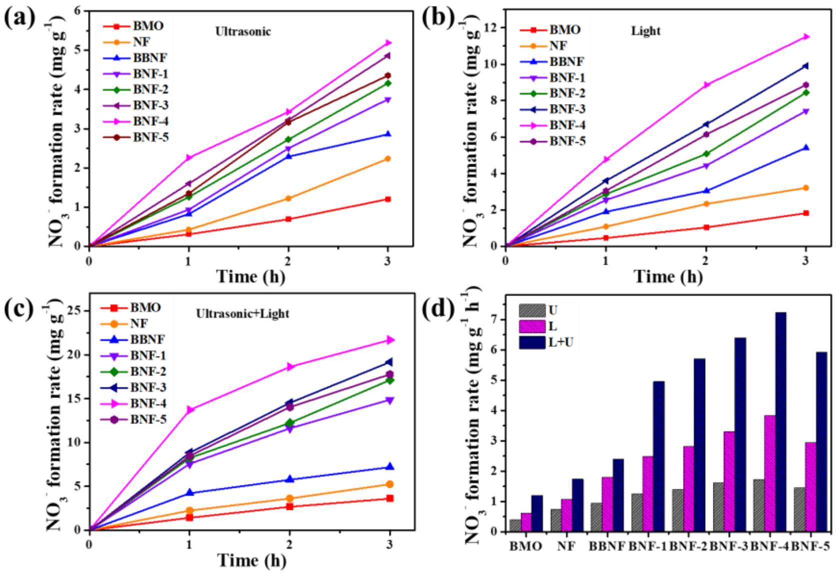

Figure S26. Photocatalytic NO_3^- yields over as-prepared samples under different condition.

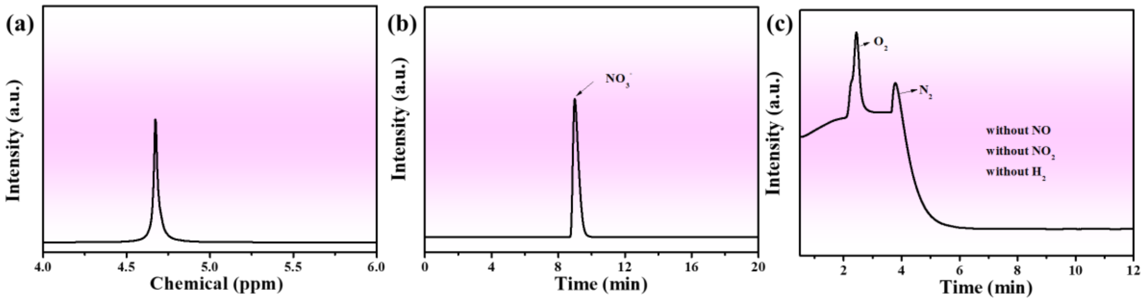

Figure S27. (a)^{1}H NMR spectra (b)The ion chromatography spectra of NO_3^- and (c)The gas chromatography spectra of piezo-photocatalytic reaction under N_2 and O_2.

Table S3. AQY of Photocatalytic NO_3^- yields over BNF-4.

Wavelength (nm)	420	450	500	550	600
NO_3^- evolution rate (μmol/g/h)	75.8718	69.1752	46.0106	26.2327	26.3474
Light intensity (W/cm^2)	0.125	0.151	0.132	0.157	0.155
Irradiation area (cm^2)	7.07	7.07	7.07	7.07	7.07
AQY (%)	0.1693	0.1198	0.0815	0.0572	0.0533

The AQY at different wavelengths was determined using monochromatic filters with a bandwidth of ± 5 nm and calculated as follows:

$$AQY\ (\%) = \frac{5 \times n(HNO_3)}{n\ (photons)} = \frac{5 \times n(HNO_3) \times m \times t \times N_A \times h \times v}{E \times S \times t} \times 100$$

Where $n(HNO_3)$ is the number of generated nitric acid molecules, n(photons) denotes the number of incident photons, m is the mass of the photocatalysts, t is the irradiation time, N_A and h are the Avogadro's constant and Planck constant, respectively, υ denotes the frequency of monochromatic light, E is the light absorption intensity, S is the irradiation area.

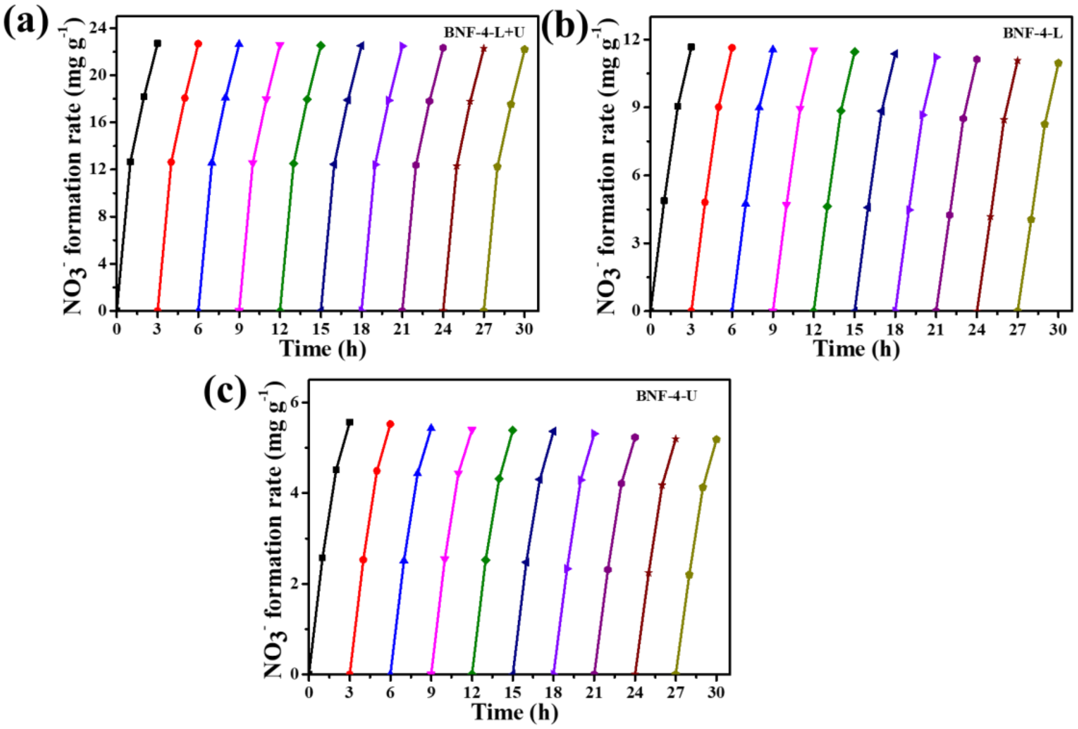

Figure S28. Cycling tests of BNF-4 (a) under light and ultrasonic, (b) under light, (c) ultrasonic.

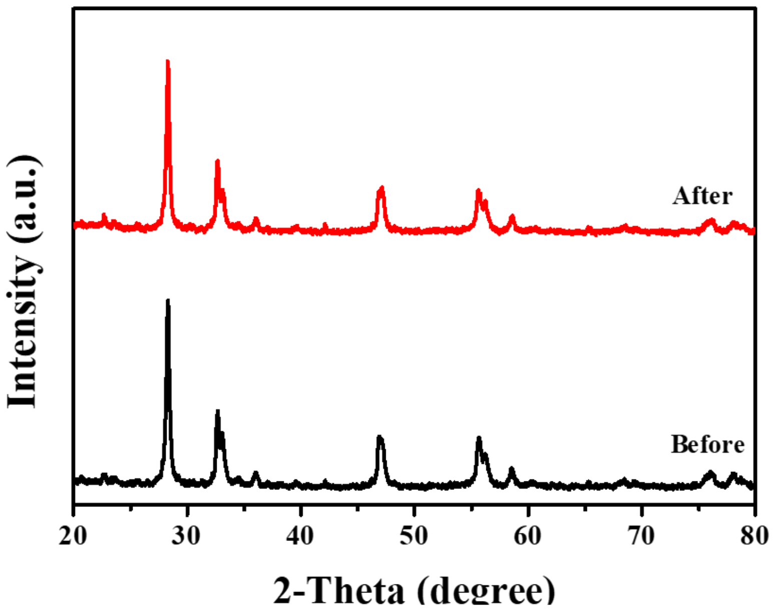

Figure S29. XRD patterns of BNF-4 after cycling ten times under light and ultrasonic

conditions.

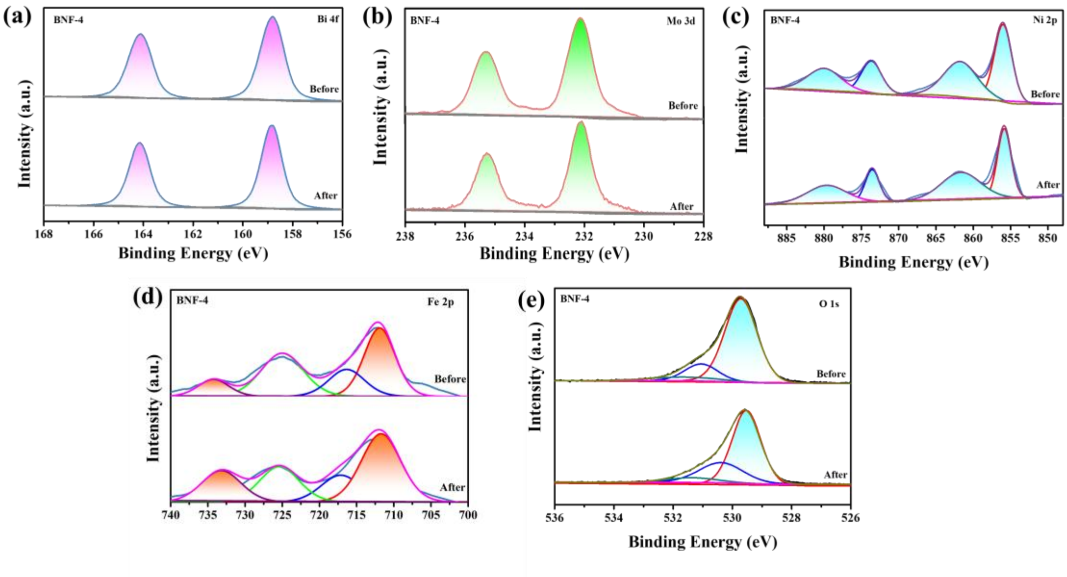

Figure S30. XPS patterns of BNF-4 after cycling ten times under light and ultrasonic conditions.

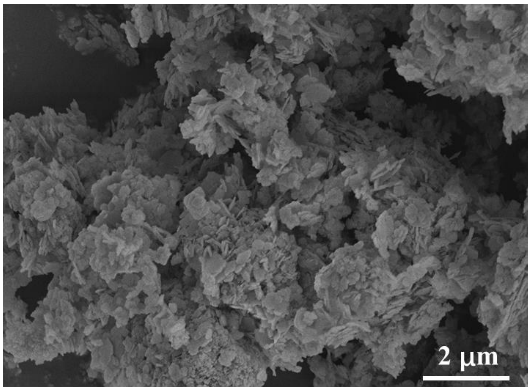

Figure S31. SEM image of BNF-4 after cycling ten times.

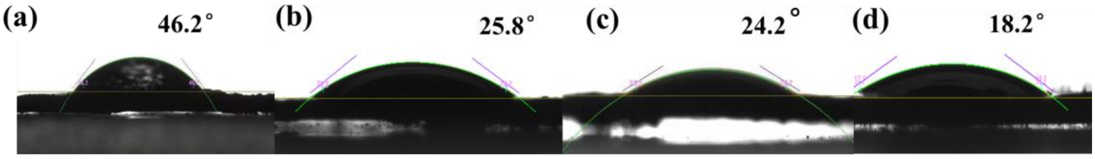

Figure S32. The contact angles of water on the surface of (a) BMO, (b) NF, (c) BBNF and (d) BNF-4.

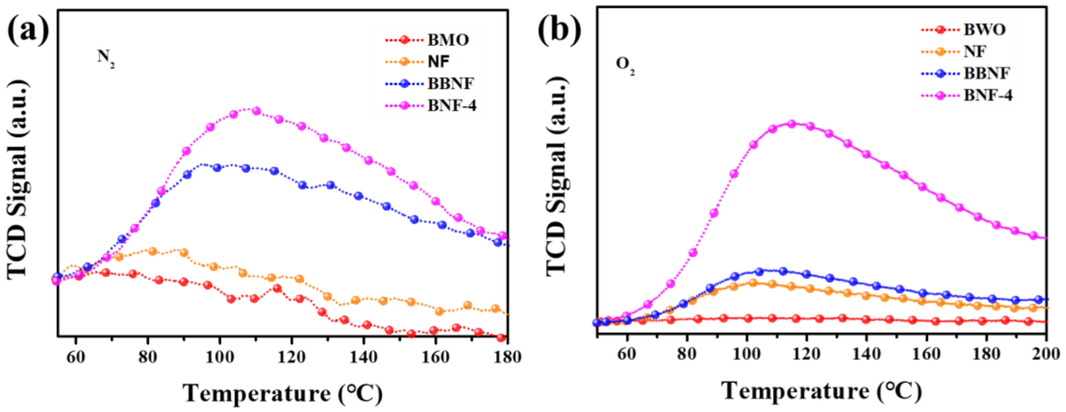

Figure S33. (a) N_2-TPD profiles of the as-prepared samples, (b) O_2-TPD profiles of the as-prepared samples.

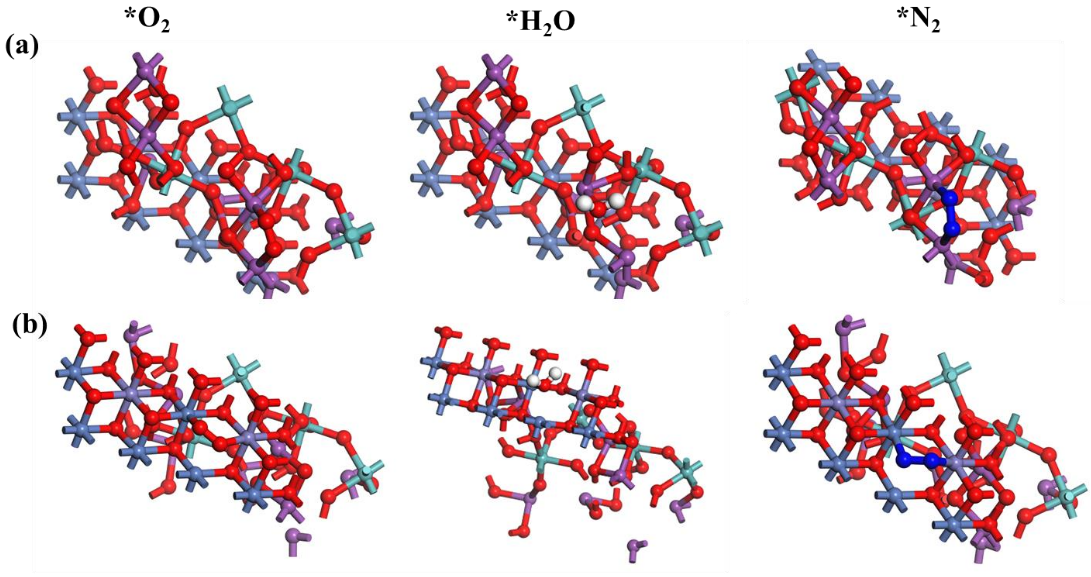

Figure S34. DFT-calculated optimized crystal structure for photocatalytic nitrogen oxidation adsorbate energy on the surface of (a)Bi_2MoO_6 and (b) NiFe-layered double hydroxide over BNF.

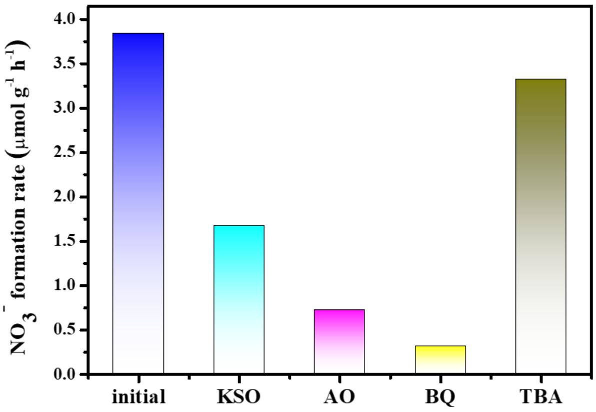

Figure S35. Photocatalytic nitrogen oxidation over BNF-4 with or without a scavenger (potassium persulfate (KSO), ammonium oxalate (AO), tert-butanol (TBA) and pbenzoquinone (BQ) scavengers for the quenching of electrons (e^-), hole (h^+) and hydroxyl radicals (•OH) and superoxide radicals ($•O_2^-$), respectively.

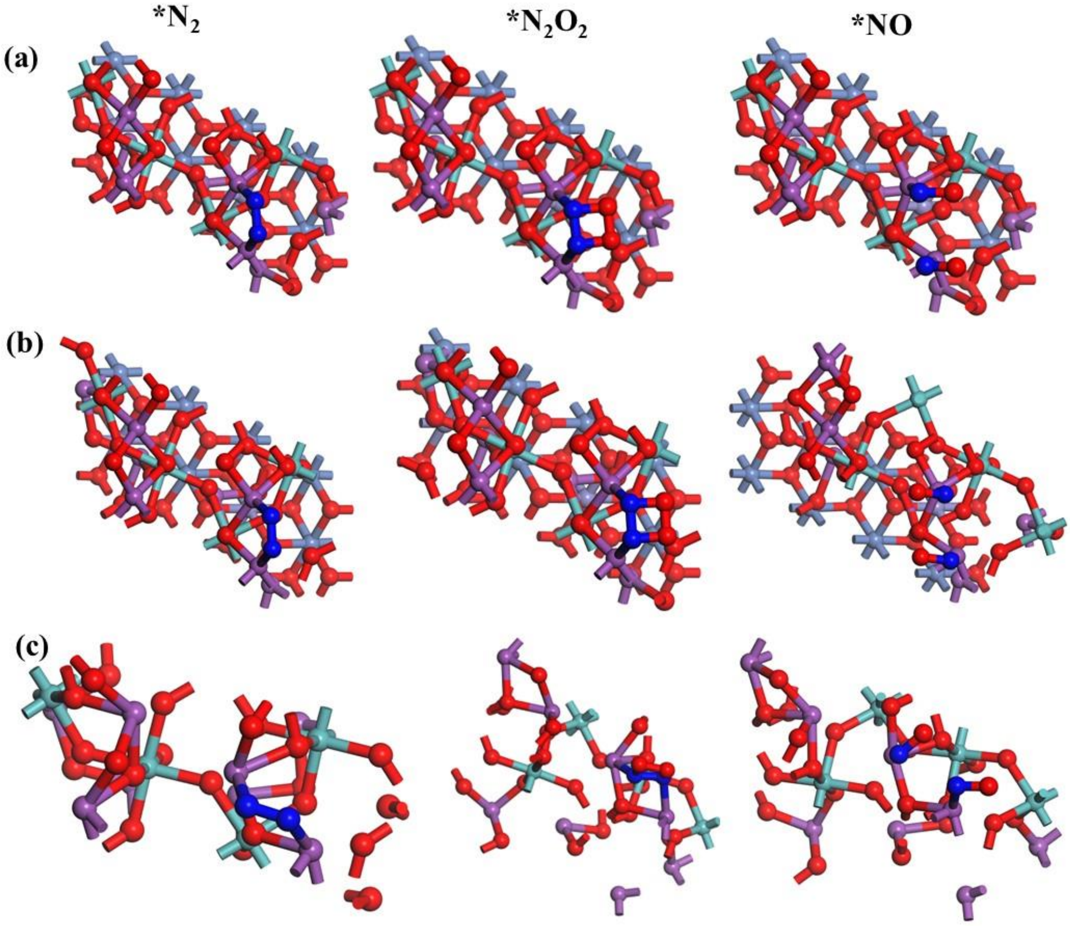

Figure S36. DFT-calculated optimized crystal structure for photocatalytic nitrogen

oxidation over (a) BNF, (b) BBNF and (c) BMO under no stress.

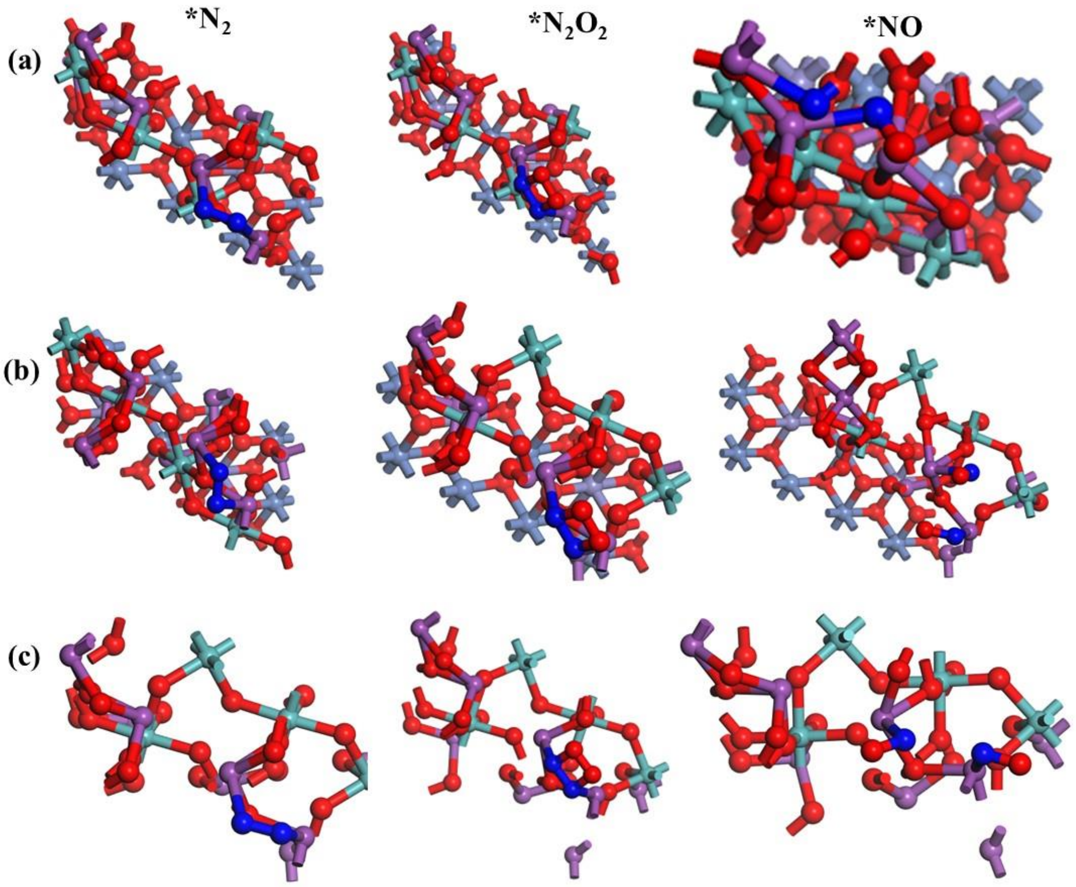

Figure S37.DFT-calculated optimized crystal structure for photocatalytic nitrogen oxidation over (a) BNF, (b) BBNF and (c) BMO under 100 MPa

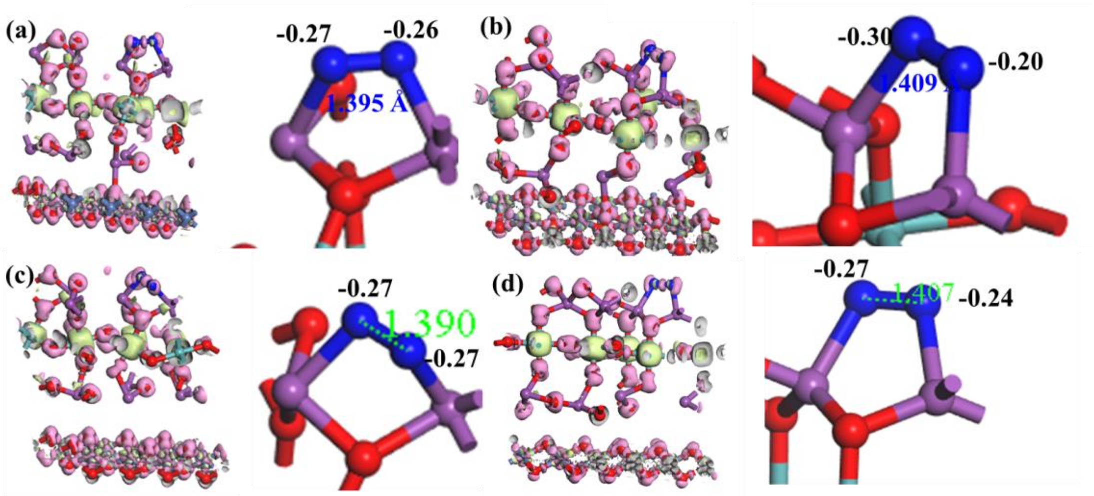

Figure S38. differential charge and charge distribution of BNF under (a) No stress, (b)100 MPa. BBNF under (c) No stress, (d)100 MPa.

Table S4 Comparison of different catalysts for photocatalytic NO_3^- yields.

Entry	Samples	Reaction condition	Evolution rate ($\mu g\ g^{-1}\ h^{-1}$)
S1	Ce-doped $W_{18}O_{49}$ nanowires	10 mg of photocatalyst, 60 mL of deionized water, 300 W Xe lamp	319.97
S2	TiO_2	1.5 g of photocatalyst, high-power UV-LED-Lamp	46.17
S3	TiO_2	0.1-g TiO_2, UV light	28.08
S4	WO_3	10 mg of photocatalyst, 60 mL of deionized water, 300 W Xe lamp	1920
S5	Pd/H-TiO_2	Catalysts (50 mg), 300 W Xenon lamp, 200 °C	204.6
S6	**This work**	**20 mg catalyst was dispersed in 50 mL of deionized water,** 300 W Xe lamp and ultrasonic condition	**7230**

[S1] J. Yang, Z. Ruan, S. Jiang, P. Xia, Q. Yang, Q. Zhang, C. Xiao, Y. Xie, *J. Phys. Chem. Lett.* **2021**, 12, 11295-11302.

[S2] A. Pashkova, B.O. Burek, J.Z. Bloh, *Catal. Sci. Technol.* **2022**, 12 2755.

[S3] S.J. Yuan, J.J. Chen, Z.Q. Lin, W.W. Li, G.P. Sheng, H.Q. Yu, *Nat. Commun.* **2013**, 4, 2249.

[S4] Y. Liu, M. Cheng, Z. He, B. Gu, C. Xiao, T. Zhou, Z. Guo, J. Liu, H. He, B. Ye, B. Pan, Y. Xie, *Angew. Chem. Int. Ed.* **2019**, 58, 731.

[S5] X. Zhang, R. Shi, Z. Li, J. Zhao, H. Huang, C. Zhou, T. Zhang, *Adv. Energy Mater.* **2022**, 2103740.
